# Achieving global perfect homeostasis through transporter regulation

**DOI:** 10.1371/journal.pcbi.1005458

**Published:** 2017-04-17

**Authors:** Yonatan Savir, Alexander Martynov, Michael Springer

**Affiliations:** 1 Department of Physiology, Biophysics and Systems Biology, Faculty of Medicine, Technion, Haifa, Israel; 2 Center for Data-Intensive Biomedicine and Biotechnology, Skolkovo Institute of Science and Technology, Moscow, Russia; 3 Department of Systems Biology, Harvard Medical School, Boston, Massachusetts, United States of America; Tel Aviv University, ISRAEL

## Abstract

Nutrient homeostasis—the maintenance of relatively constant internal nutrient concentrations in fluctuating external environments—is essential to the survival of most organisms. Transcriptional regulation of plasma membrane transporters by internal nutrient concentrations is typically assumed to be the main mechanism by which homeostasis is achieved. While this mechanism is homeostatic we show that it does not achieve global perfect homeostasis—a condition where internal nutrient concentrations are completely independent of external nutrient concentrations for all external nutrient concentrations. We show that the criterion for global perfect homeostasis is that transporter levels must be inversely proportional to net nutrient flux into the cell and that downregulation of active transporters (activity-dependent regulation) is a simple and biologically plausible mechanism that meets this criterion. Activity-dependent transporter regulation creates a trade-off between robustness and efficiency, i.e., the system's ability to withstand perturbation in external nutrients and the transporter production rate needed to maintain homeostasis. Additionally, we show that a system that utilizes both activity-dependent transporter downregulation and regulation of transporter synthesis by internal nutrient levels can create a system that mitigates the shortcomings of each of the individual mechanisms. This analysis highlights the utility of activity-dependent regulation in achieving homeostasis and calls for a re-examination of the mechanisms of regulation of other homeostatic systems.

## Author summary

Homeostasis, the ability to maintain relatively constant internal conditions in the face of fluctuating environments, is fundamental to many biological processes. In nutrient homeostasis, a model homeostatic system, homeostasis is typically thought to be achieved through negative feedback regulation of the plasma membrane transporters synthesis by intracellular nutrient levels. Here, we first derive the general conditions that can achieve global perfect homeostasis in a simple uptake system. We found that this condition can be satisfied by the ubiquitous but less studied mechanism of *activity-dependent* transporter downregulation. If transporter downregulation is dependent on nutrient uptake rates, i.e., activity-dependent downregulation, the system in principle can achieve homeostasis in any external environment. Activity-dependent and internal regulation can synergize to achieve homeostasis across a wide set of conditions at minimal energetic cost. Activity-dependent downregulation is likely to play a role in many diverse homeostatic systems.

## Introduction

Cells must maintain relatively constant internal concentrations of nutrients even though the supply of nutrients from the environment can fluctuate wildly, a process called nutrient homeostasis [1,2]. In microorganisms, errors in nutrient homeostasis can have dramatic effects on growth, since low internal nutrient concentrations limit growth, while excessive internal nutrient concentrations can be toxic [3,4]. In mammalian cells, nutrient uptake, cell growth, and proliferation are controlled by the overlapping signaling pathways [5,6] and defects in nutrient regulation play a role in the pathogenesis of diseases such as cancer and diabetes [1,7–9]. Nutrient homeostasis is a major determinant of both organismal and cellular fitness.

There are two axes that are important for homeostasis. The first axis is the robustness of homeostasis: the more robust the homeostasis, the smaller the change in internal nutrient concentration for a given change in external nutrient concentration. The limit of robust homeostasis is when the internal nutrient concentration is completely insensitive to the external nutrient concentration, a condition we refer to as 'perfect' homeostasis. The second axis is the range of homeostasis. The wider the range of homeostasis, the large the range of external nutrient concentrations over which the system achieves a given robustness of homeostasis. Global homeostasis occurs when the system is homeostatic regardless of the external nutrient concentration. In this work, we solve for conditions that achieve global perfect homeostasis.

**Fig 1:**
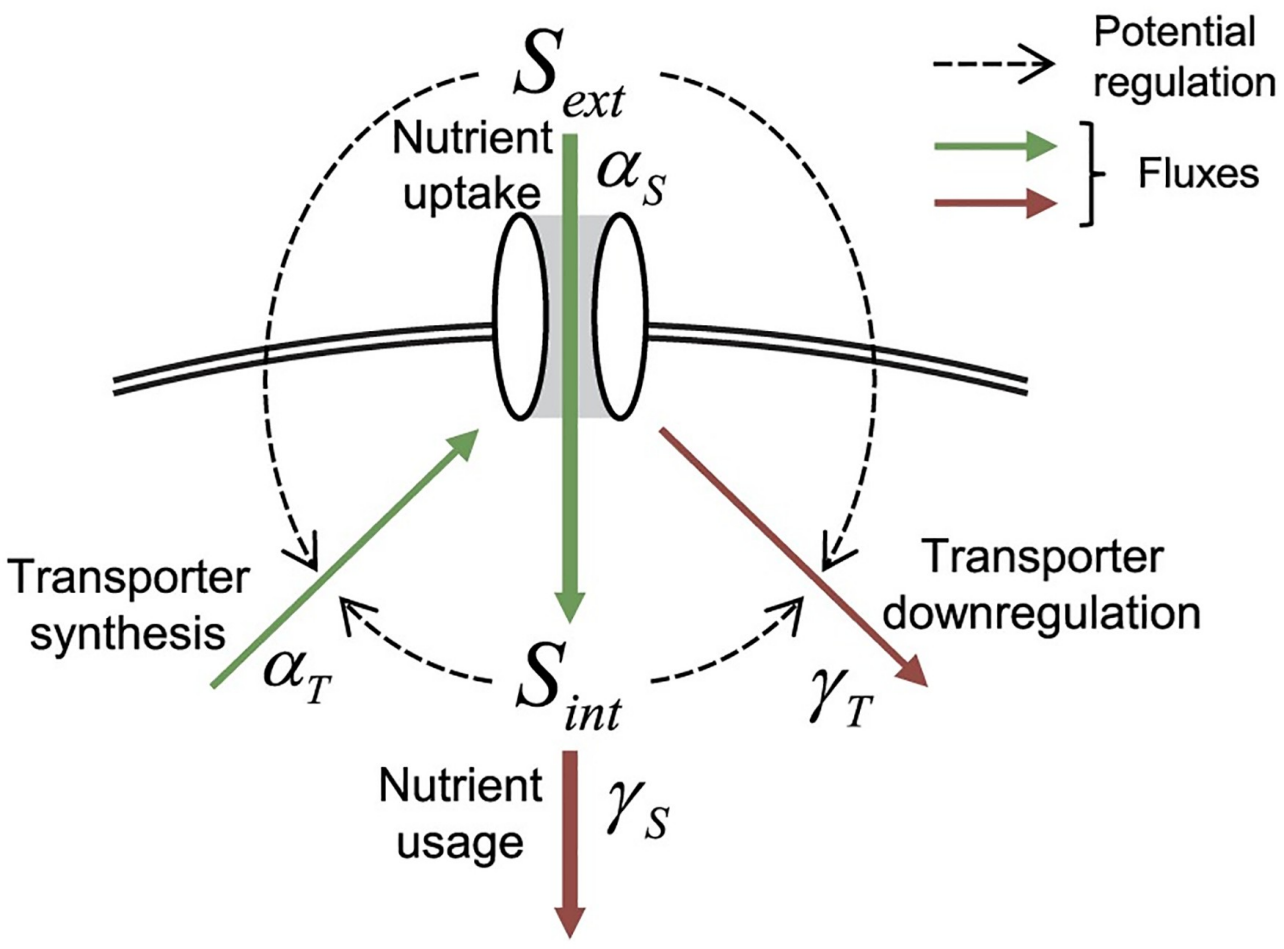
Homeostasis in a general uptake system. Schematic of a general nutrient uptake system. The system is comprised of a transporter that can transport nutrient across a plasma membrane, i.e. it converts external nutrient (*S*_*ext*_) to internal nutrient (*S*_*int*_). Transporters are synthesized (thin green arrow) and downregulated (thin red arrow). *α_T_* denotes transporter synthesis, *γ_T_* transporter downregulation, and *γ_s_* internal nutrient usage. All these terms represent fluxes. Regulation of synthesis can be achieved by a number of mechanisms, e.g. regulation of transcription or trafficking. Regulation of downregulation can be achieved by a number of mechanisms, e.g. regulation of transporter activity or transporter degradation/dilution. Nutrients are imported (thick green arrow) and used (think red arrow). *S*_*int*_ [3,4,11,27] and *S*_*ext*_ [19] can in principle be sensed and therefore could affect transporter synthesis and downregulation (dotted lines).

There are many examples of biological systems that exhibit homeostasis [10–14]. In general, homeostasis can arise from fine tuning kinetic parameters or from structural properties of the regulating network [1,2,12,14]. Achieving nutrient homeostasis requires cellular circuitry that is able to sense nutrient levels and then regulate uptake and/or usage accordingly. All nutrient homeostatic systems need a plasma membrane transporter that allows passage of the nutrient through the plasma membrane. The majority of nutrient homeostatic systems share a common architecture where the synthesis of this plasma membrane transporter is under the regulation of nutrient levels (Fig 1) [3,4,11]. This regulation is a negative feedback system such that when nutrient concentrations are low, transporter synthesis is increased and when nutrient synthesis is high transporters, synthesis is decreased [1,5,6,11]. In eukaryotes, this type of regulation has been demonstrated for metal ions [10,15–18], sugars [1,2,19,20], phosphate [3,4,21], and amino acid transport [6,22–24]. While the mechanistic details of this design can vary, e.g. regulation of synthesis through transcription [1,7–9,25] or trafficking [10,26], regulation of transporter synthesis is typically assumed to be the critical factor in nutrient homeostasis. It has been shown that this negative feedback regulation makes nutrient homeostasis more robust and this robustness depends on the sensitivity of the transporter synthesis rate to nutrient levels [1,2,12–14].

A second and equally widespread motif in homeostatic systems is post-translational downregulation of transporters [28–32]. As with regulation of synthesis, the mechanistic details of this design can vary; e.g., plasma membrane transporters can be inactivated by modification, sequestrations, or degradation. In yeast, this mode of regulation has been demonstrated for many different homeostatic systems including ion transporters such as zinc, copper, and iron [33–35]; sugar transporters such as glucose and maltose transporters [25,36]; a phosphate transporter [37]; and amino acid transporters [25,38]. This mechanism is usually considered a stress response to an extreme change in nutrients [39]. Indeed, the response to extreme changes is a homeostatic process. Transporter downregulation has been less well studied than transcriptional regulation of transporter synthesis. In the best-characterized systems, transporter downregulation is mediated by ubiquitination [25,40–42] but the theoretical cost and benefits of using transporter downregulation to achieve homeostasis are not understood.

Mathematically, regulation by transporter synthesis and transporter downregulation are interconvertible at steady-state; i.e., adding a term to the transporter synthesis flux (thin green arrow in Fig 1) and adding 1 over that term to the transporter downregulation flux (thin red arrow in Fig 1) yields the same steady state solution. Yet, biological realizations of some mathematical terms are not easily achieved. Hence, these two forms of regulation may not be biologically interconvertible. We determined that if transporter levels are inversely proportional to flux through the plasma membrane transporters, global perfect homeostasis is achieved. Flux sensing is distinct from the internal and external nutrient sensing that is typically considered to regulate nutrient homeostatic systems. Work from the Heinemann lab [43,44] has shown that flux sensing is used for the regulation of some intracellular metabolism. We showed that flux sensing based homeostasis can be easily achieved if active transporters are downregulated. We will refer to this as activity-dependent downregulation of transporters. Given that both transporter synthesis and downregulation are regulated in most nutrient homeostatic systems, we sought to determine the potential trade-offs of each architecture [45–48]. The combination of activity-dependent downregulation and regulation through synthesis makes a more efficient system than either mechanism alone.

## The general criterion for global perfect homeostasis

To define homeostasis we consider a dynamical system that has an output, e.g. the internal nutrient concentration (*S*_*int*_), which depends on other variables such as external nutrient concentration (*S*_*ext*_) and transporter concentration (*T*). The output of the system achieves *global perfect homeostasis* with respect to a specific variable if the stationary concentration of the output is invariant to perturbations in this variable. Formally, for the example of nutrient transport, *S*_*int*_ would achieve global perfect homeostasis with respect to *S*_*ext*_ if *S*_*int*_ has a steady state such, $S_{int}^{ss}$, such that $\frac{\partial S_{int}^{ss}\left(Sext,T_{}^{ss}(Sext)\right)}{\partial Sext}=0$ for every value of *S*_*ext*_. Some systems may exhibit perfect homeostasis for a range of *S*_*ext*_ values; we refer to this as *local homeostasis*. Additionally, to quantify the dependence of the $S_{int}^{SS}$ on *S*_*ext*_, we defined a unitless parameter that is the steady state value of *S*_*int*_ for a given *S*_*ext*_ normalized by the maximal steady state value of *S*_*int*_ over the range of relevant *S*_*ext*_, $r(S_{ext})=\frac{S_{int}^{ss}\left(Sext,T_{}^{ss}(Sext)\right)}{\underset{S_{ext}=[0,\infty )}{\text{max}}S_{int}^{ss}\left(Sext,T_{}^{ss}(Sext)\right)}$. We will refer to, *r*, as the robustness of homeostasis. A system achieves global perfect homeostasis when *r* = 1 for every *S*_*ext*_. When the system is not perfectly homeostatic, *r* can be any value between zero and one and this value can change as a function of *S*_*ext*_. Biological systems that approach this limit of perfect homeostasis, i.e. *r* is close to 1, are often still considered homeostatic although there is no standard value for *r* which segments between whether or not a system is considered homeostatic [10]. In this work, we look for the biological circuitries that achieve global perfect homeostasis. Note that homeostasis as we define it is a property of the steady state concentration; during transitions between different steady states, the homeostatic output can transiently change.

We first we sought to determine all conditions that could lead to global perfect homeostasis in a general uptake system (Fig 1). Our system is composed of an external nutrient (*S*_*ext*_), an internal nutrient (*S*_*int*_), and a plasma membrane transporter (*T*) (Fig 1). Transporters allow nutrient to pass into the cell through the plasma membrane and can be both synthesized and destroyed. While there are more molecular players, e.g., mRNA and translocation machinery, this system encapsulates the key biological variables while subsuming the rest of the players into the parameters. We use the following notation convention: Greek symbols denote fluxes (with units of concentration/time), the symbol *k* denotes rate constants (with units of 1/time or 1/concentration/time depending on the reaction order), and *u* denotes nutrient flux per transporter. This system (Fig 1) can be described by two ordinary differential equations,

$$\begin{array}{ll} {\dot{S}}_{int} & =T\cdot u(S_{ext},S_{int})-\gamma_{S}(S_{ext},S_{int})\\ \dot{T} & =\alpha_{T}(S_{ext},S_{int},T)-k_{\gamma_{T}}(S_{ext},S_{int})\cdot T, \end{array}$$ (1)

where *u* is the rate of nutrient uptake per transporter, *γ*_*s*_ is the nutrient usage flux, *α*_*T*_ is the transporter synthesis flux, and $k_{\gamma_{T}}$ is the transporter downregulation rate. We made the standard simplifying assumption that transporter downregulation flux is linearly proportional to transporter levels, $\gamma_{{}_{T}}=k_{\gamma_{T}}(S_{ext},S_{int})\cdot T$ [49–51]. In theory, the system could be further generalized by making *u*, *γ*_*s*_, and $k_{\gamma_{T}}$ arbitrary functions of *T*, but this is not supported by the biology of any of the commonly studied nutrient uptake systems. Simplified versions of this system, such as Eq (1), have been used to show that internal nutrient-dependent regulation of transporter synthesis can be homeostatic and thereby has provided a rationale for the ubiquity of this architecture [10].

To define the necessary and sufficient conditions for global perfect homeostasis we applied the method of Steuer et al. [14]. The conditions that are necessary and sufficient for homeostasis of this system are (S1 Appendix, sections I, II):

$$\frac{\partial_{S_{ext}}\left(k_{\gamma_{T}}(S_{ext},S_{int})/\alpha_{T}(S_{ext},S_{int},T)\right)}{k_{\gamma_{T}}(S_{ext},S_{int})/\alpha_{T}(S_{ext},S_{int},T)\cdot (1-\frac{T\cdot \partial_{T}\alpha_{T}(S_{ext},S_{int},T)}{\alpha_{T}(S_{ext},S_{int},T)})}=\frac{\partial_{S_{ext}}u(S_{ext},S_{int})}{u(S_{ext},S_{int})}-\frac{\partial_{S_{ext}}\gamma_{s}(S_{ext},S_{int})}{\gamma_{s}(S_{ext},S_{int})}.$$ (2)

This relationship is complicated and it is hard to imagine the regulatory interactions that would allow a biological instantiation of this general system. But Eq (2) does yield the insight that global perfect homeostasis is achieved by regulating transporters levels (*T*) such that they compensate for the change in usage rate (*γ_s_*) or uptake per transporter (*u*). In the following sections, we will constrain this general system; this will reduce Eq (2) to a condition that has a clear biological interpretation.

## Flux-dependent regulation can achieve global perfect homeostasis

### A reduced system

We sought to determine special cases of the general uptake system described in Eq (1) where the resulting homeostatic criterion is achievable by biologically plausible mechanisms. We started with the following biologically reasonable and standard assumptions: 1) there is little or no evidence for transporter levels directly affecting transporter synthesis, *α*_*T*_(*S*_*ext*_, *S*_*int*_); 2) *S*_*ext*_ negligibly affect nutrient usage, *γ*_*S*_(*S*_*int*_); and 3) internal nutrients negligibly affect nutrient uptake per transporter, *u*(*S*_*ext*_). Under these simplifying assumptions, Eq (1) reduces to:

$$\begin{array}{ll} {\dot{S}}_{int} & =T\cdot u(S_{ext})-\gamma_{S}(S_{int})\\ \dot{T} & =\alpha_{T}(S_{ext},S_{int})-k_{\gamma_{T}}(S_{ext},S_{int})\cdot T \end{array}.$$ (3)

**Fig 2:**
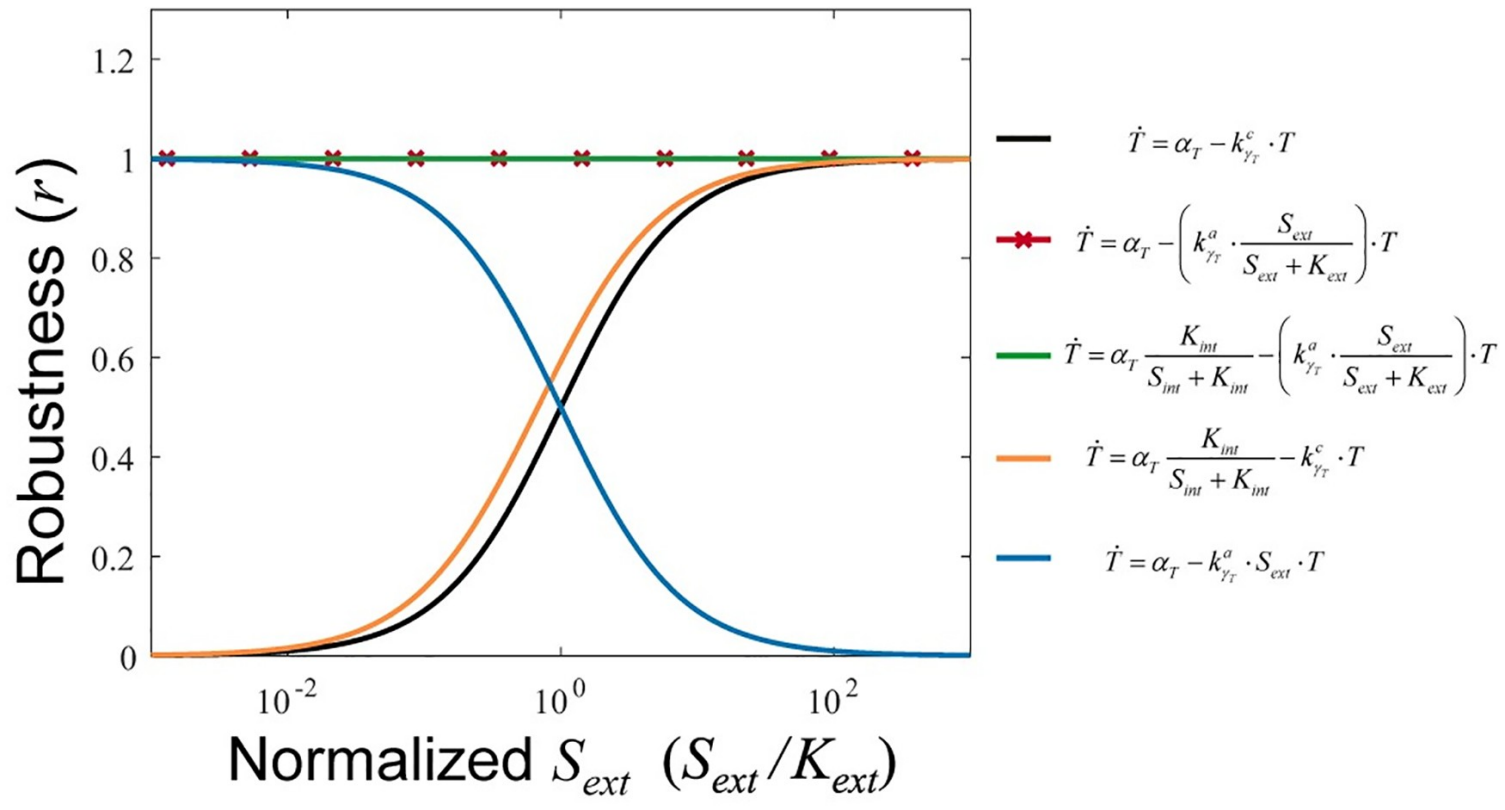
Homeostasis by activity-dependent transporter downregulation. The steady-state internal nutrient concentration for a range of external nutrient concentrations is plotted for six different instantiation of the system in Eq (3): no transporter regulation (black), flux-based transporter downregulation (red with x's), internal nutrient based repression of transporter synthesis (orange), linear external nutrient based transporter downregulation (blue), flux-based transporter downregulation plus internal nutrient based repression of transporter synthesis (green). This illustrates the criterion from Eq (4), which states that the system can only achieve global perfect homeostasis (red and green lines) when flux-based transporter downregulation ($k_{\gamma_{T}}$) is combined with an arbitrary function of the internal nutrient concentration (*S*_*int*_).

The criterion for homeostasis, Eq (2) (S1 Appendix, section II), reduces to:

$$\frac{k_{\gamma_{T}}(S_{ext},S_{int})}{\alpha_{T}^{}(S_{ext},S_{int})}\propto u(S_{ext})\cdot func(S_{int})$$ (4)

where *func*(*S*_*int*_) is *a general* function that depends solely on *S*_*int*_ (and could also be constant) (Fig 2). This condition states that any system in which the regulation of the transporter does not explicitly depend on *S*_*ext*_ cannot provide global perfect homeostasis. Moreover, as transporter levels at steady state are given by $\alpha_{T}/k_{\gamma_{T}}$, this criterion is satisfied when the transporter level, at steady state, is inversely proportional to the nutrient uptake rate. When this condition is met, the solution for the steady state value of *S*_*int*_ is given by solving

$$0=func(S_{int})-\gamma_{s}(S_{int}).$$ (5)

While, this condition does not guarantee a steady state solution for *S*_*int*_, when a solution exists, it is *independent* of *S*_*ext*_.

### Biological plausibility

**Fig 3:**
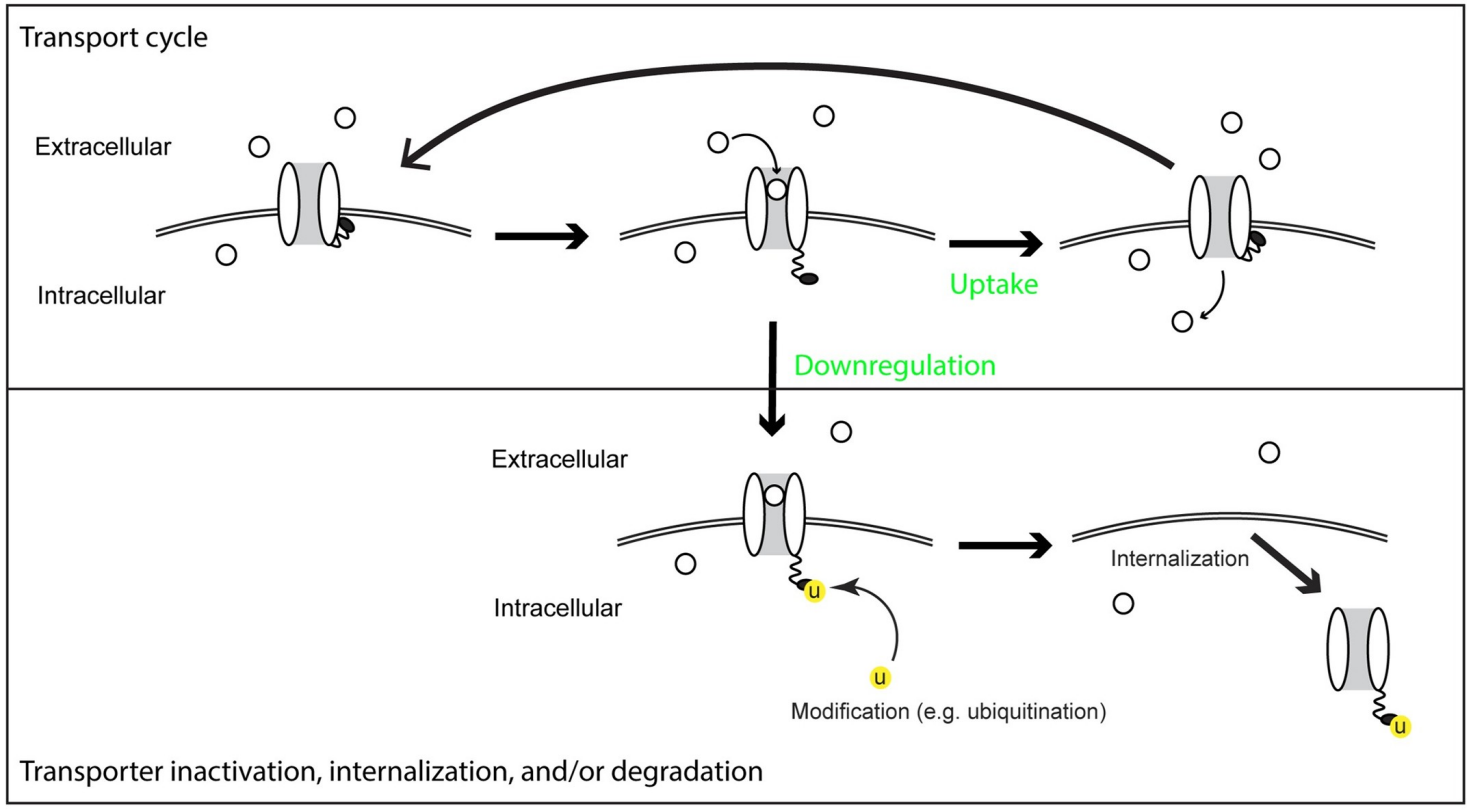
A molecular mechanism that can achieve activity-dependent transporter downregulation. A nutrient molecule (white circle) binds a transporter (a white pair of ovals) to form a nutrient-transporter complex. This complex then undergoes a conformational change. The complex can then either: 1) Uptake—nutrient is released into the cell; the transporter finishes the uptake cycle by returning to its ground state, or 2) Downregulation—the transporter is modified leading directly and/or indirectly to its inactivation. Because uptake and downregulation involve the same molecular species the rates or these two processes are automatically proportional.

Is there a biological mechanism that can satisfy Eq (4) by making transporter downregulation proportional to nutrient uptake per transporter, i.e. $k_{\gamma_{T}}(S_{ext},S_{int})\propto u(S_{ext})$? This condition corresponds to the requirement that only transporters that are actively transporting have the potential to be downregulated. We postulated that a simple biological scheme could achieve activity-dependent downregulation and thereby satisfy Eq (4). This scheme is composed of a standard transporter cycle, where: 1) external nutrient binds to a transporter, 2) the transporter undergoes a conformational change allowing the nutrient to be released on the opposite side of the membrane, and 3) the transporter returns to its original conformation. In addition to this core system, we added an enzyme that recognizes and modifies only the nutrient bound conformation of the transporter (Fig 3). The modified transporter is downregulated. As long as the process is irreversible, direct and indirect inactivation are equivalent. Indeed, this system almost trivially couples the uptake and downregulation rate making them directly proportional (Fig 3 and S1 Appendix, section IV).

Two studies directly link uptake and downregulation [52,53]. In both cases, nutrient uptake leads to a conformational change in the transporter that is then ubiquitinylated in a manner analogous to our scheme in Fig 3. This basic scheme is also supported by a series of crystal structures that show the conformational changes that occur upon nutrient binding [54,55].

While we only know of two proven examples for transporters, there are many examples of activity-dependent downregulated receptors. For example, ligand-dependent modification and downregulation is a core feature of G protein-coupled receptors and is required for ligand-mediated desensitization [56–62]. In bacteria, methyl-accepting chemotaxis receptors are modified in a ligand-dependent manner and this modification affects their sensing [63,64]. This activity-dependent methylation was demonstrated to be critical for robust adaptation [65] a process that is similar to homeostasis. While these examples involve receptors, transports share many features with receptors. Nutrient sensors and transporters are high related [66–71]. Multiple transporters have both transporting and signaling functions [72–78] and point mutations can interconvert receptors and transporters [79]. In some cases, receptor-mediated endocytosis [80–82] can even be considered a hybrid of transport and downregulation. Furthermore, transporters are modified and internalized in a manner that is very similar to receptors. Many transporters undergo internalization and degradation in a ubiquitin-dependent manner.

Together, these examples argue that the proposed activity-dependent mechanism is not just mathematically possible but likely ubiquitous. The paucity of examples is likely due to a lack of experiments that have been performed in a manner such that activity-dependent regulation would have been observed. Indeed, changing the external nutrient concentration can stimulate transporter degradation [21,28,30,32–34] or inactivation [30,37,52,83,84] consistent with a ubiquitous role of activity-dependent regulation.

### Activity-dependent regulation in non-idealized conditions

When activity-dependent downregulation is the only form of transporter downregulation, global perfect homeostasis can be achieved. But, in real systems, dilution from cell growth and basal protein degradation will always contribute to transporter downregulation. To isolate the impact of dilution and protein degradation on global perfect homeostasis we considered the following minimal system:

$$\begin{array}{ll} {\dot{S}}_{int} & =k_{cat}\cdot T\cdot \frac{S_{ext}}{S_{ext}+K_{ext}}-k_{\gamma_{S}}\cdot S_{int}\\ \dot{T} & =\alpha_{T}-\left(k_{\gamma_{T}}^{c}+k_{\gamma_{T}}^{a}\cdot \frac{S_{ext}}{S_{ext}+K_{ext}}\right)\cdot T \end{array}.$$ (6)

**Fig 4:**
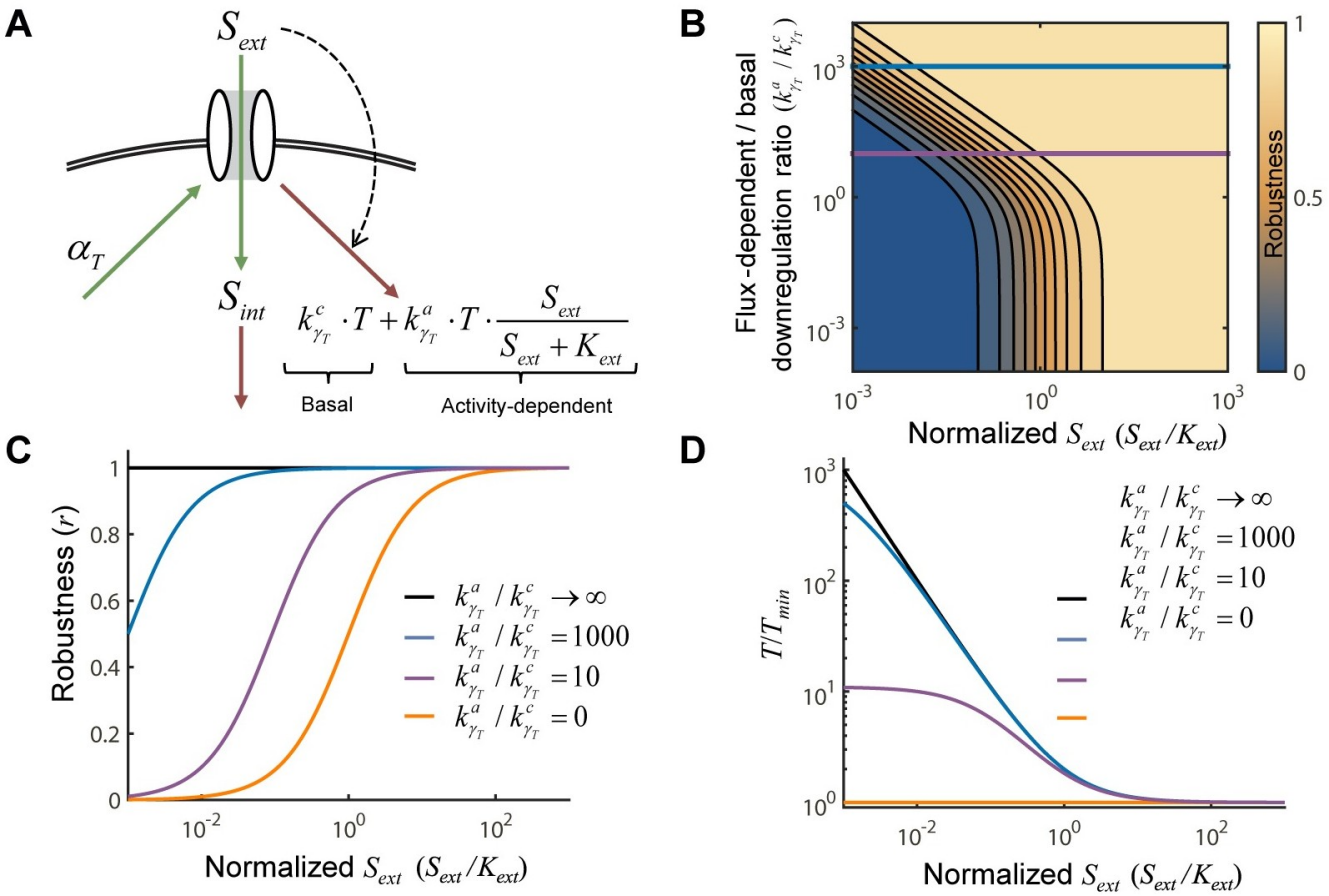
Global perfect homeostasis is limited by dilution and basal degradation. **(A)** Schematic of a system where transporter degradation occurs by two mechanisms: (1) activity-dependent downregulation (rate constant of $k_{\gamma_{T}}^{a}$) and (2) dilution and basal degradation (rate constant $k_{\gamma_{T}}^{c}$). **(B)** Robustness, *r*, of the system from (A) given different ratios of $k_{\gamma_{T}}^{a}$ to $k_{\gamma_{T}}^{c}$. The blue and magenta cross-sections correspond to the blue and magenta curves in (C). **(C)** In the limit where $k_{\gamma_{T}}^{a}$ goes to zero, *S*_*int*_ tracks *S*_*ext*_ (orange). In the limit where $k_{\gamma_{T}}^{c}$ goes to zero, the *S*_*int*_ is independent of the *S*_*ext*_ (black). **(D)** Transporter levels as a function of *S*_*ext*_ for the same four ratios of $k_{\gamma_{T}}^{a}$ to $k_{\gamma_{T}}^{c}$ as in (C). As $\frac{k_{\gamma_{T}}^{a}}{k_{\gamma_{T}}^{c}}\to \infty$, *T* changes inversely with *S*_*ext*_ which allows the system to achieve homeostasis.

In this case, *α*_*T*_ is constant, and the uptake per transporter has a standard Michaelian form, $u=k_{cat}\frac{S_{ext}}{S_{ext}+K_{ext}}$. $k_{\gamma_{T}}^{a}$ is maximal activity-dependent downregulation rate constant and $k_{\gamma_{T}}^{c}$ is the combined rate constant for all other downregulation processes (Fig 4A). We additionally assumed that nutrient uptake is Michaelian, while this was not essential, it is the standard assumption for nutrient uptake kinetics.

Given that basal degradation and dilution are not homeostatic, the robustness of homeostasis, as quantified by our robustness metric, *r*, depends on the relative magnitude of $k_{\gamma_{T}}^{a}$ and $k_{\gamma_{T}}^{c}$ (Fig 4B). As $k_{\gamma_{T}}^{a}/k_{\gamma_{T}}^{c}$ increases, the system, Eq (6), becomes more robust to changes in *S*_*ext*_ (Fig 4B and 4C). In the limit where activity-dependent downregulation dominates, $k_{\gamma_{T}}^{a}\;>>\;k_{\gamma_{T}}^{c}$, homeostasis is achieved (S1 Appendix, section V); in the limit where the degradation and dilution dominates, $k_{\gamma_{T}}^{a}\;<<\;k_{\gamma_{T}}^{c}$, *S*_*int*_ tracks *S*_*ext*_. While *S*_*int*_ is robust to changes in *S*_*ext*_ when $k_{\gamma_{T}}^{a}\;>>\;k_{\gamma_{T}}^{c}$, transporter levels are not. Instead, *T* tracks *S*_*ext*_ and serves as the latent variable that allows the system to be robust; *T* adapts to keep the uptake rate, $k_{cat}\cdot T\cdot \frac{S_{ext}}{S_{ext}+K_{ext}}$, constant (Fig 4D). The decrease in robustness when basal degradation dominates is mirrored by a decrease in the sensitivity of the *T* to *S*_*ext*_ (Fig 4D).

### Alternate mechanism of flux sensing

**Fig 5:**
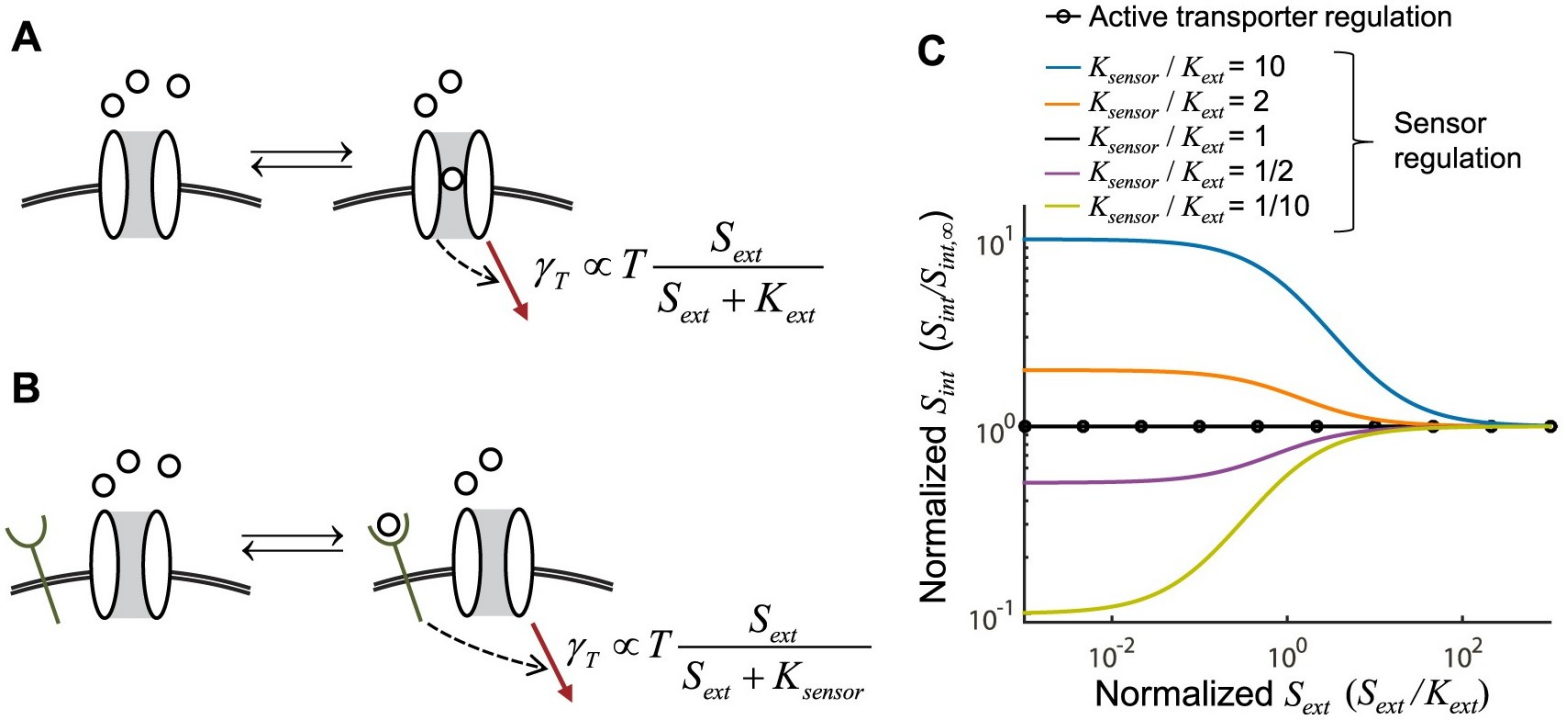
Flux-based regulation through an external nutrient sensor is more constrained than activity-dependent downregulation. **(A)** Downregulation that depends on active transporters. **(B)** Downregulation that depends on an external sensor. **(C)** Sensor based downregulation can achieve global homeostasis only if the affinities of the sensor and the transporter are comparable, *K*_*Sensor*_ ≈ *K*_*ext*_. Normalized *S*_*int*_ is similar to our robustness metric, *r*, except that it is normalized to *S*_*int*_ at high *S*_*ext*_ instead of the maximal *S*_*int*_ at any *S*_*ext*_.

We wished to explore whether other common forms of regulation could achieve global perfect homeostasis. The criterion of Eq (4) could also be satisfied by a sensor that directly measures the nutrient flux (Fig 5A) or a sensor with identical binding kinetics as the transporter (Fig 5B). This sensor could then regulate transporter synthesis or downregulation. Recent theoretical and experimental works from Kotte et al. and Kochanowski et al. have described the existence and role that flux sensing can play in metabolic regulation [43,44] and some nutrient systems contain external nutrient sensors [67]. If either of these sensors led to the downregulation of nutrient transporters it would be functionally equivalent to activity-dependent downregulation. In fact, the enzyme that modifies the nutrient bound transporter in Fig 3 is effectively acting as a flux sensor. But, both of these other mechanisms would require an extra level of regulation to normalize for the number of sensor molecules. In the case of an internal flux sensor, the activity of the sensor depends on the total nutrient flux, *T*⋅*u*(*S*_*ext*_). Simple molecular interactions between the transporter and sensor would lead the downregulation rate of the transporter to depend on the activity of the transporters multiply the number of transporters, i.e. square of the transporter concentration, *u*(*S*_*ext*_)⋅*T*^2^.

In the case of an external sensor, the activity of the sensor depends on binding the external nutrients, so the transporter downregulation will have the form $k_{\gamma_{T}}(S_{ext},S_{int})\propto T\frac{S_{ext}}{S_{ext}+K_{sensor}}$. Since uptake has the form of $k_{\gamma_{T}}(S_{ext},S_{int})\propto u(S_{ext})$, this mode of regulation will only achieve homeostasis when the sensor's Michaelis constant is close to transporter's Michaelis constant (Fig 5C; S1 Appendix, section VI). As these constants deviate, the system loses the ability to be robust to changes in external nutrient concentration (Fig 5C). Therefore, while all three mechanisms are biologically feasible, we believe activity-dependent downregulation will be the most common.

## Comparison and combination of activity-dependent downregulation and internal nutrient sensing

**Fig 6:**
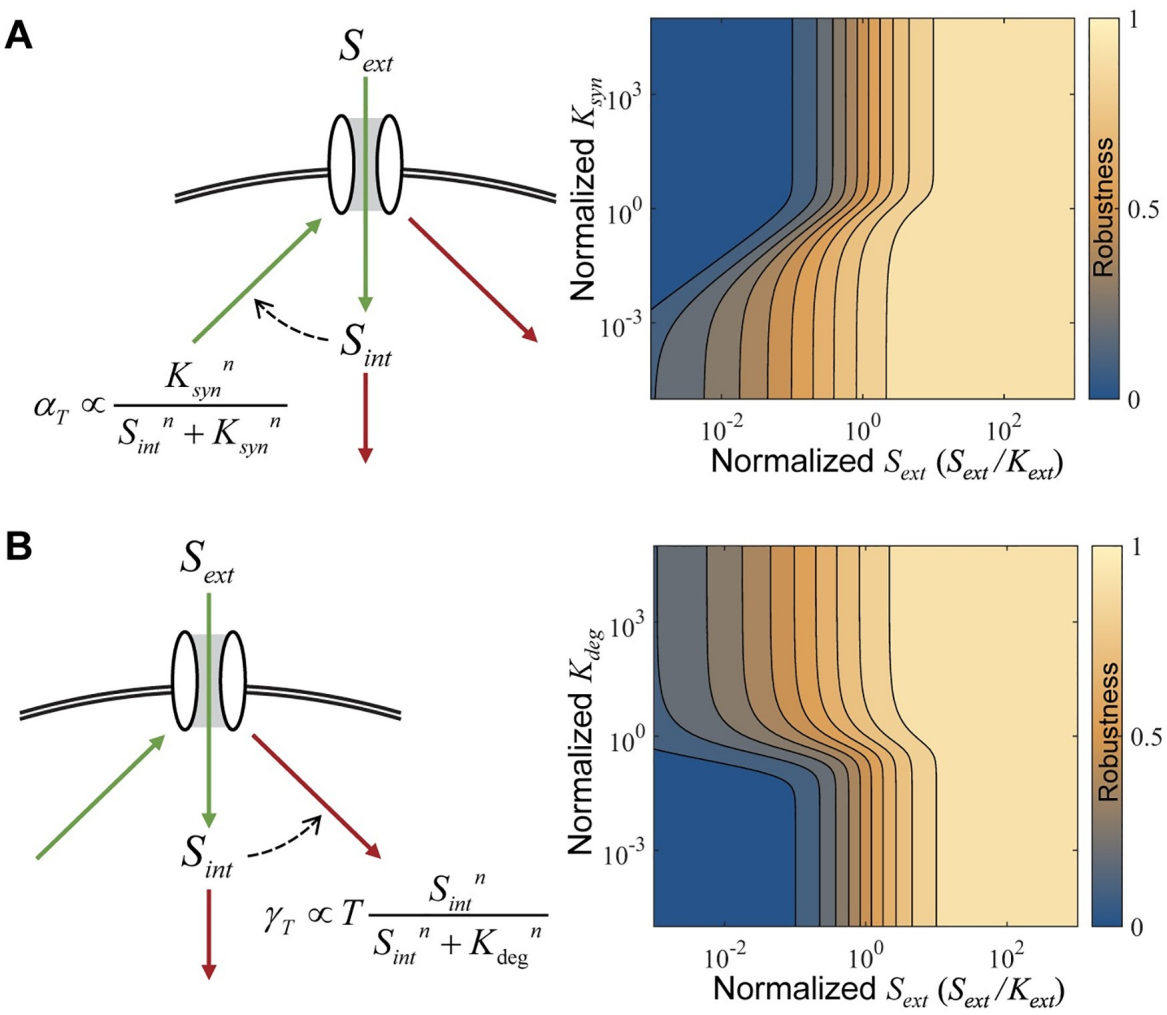
Regulation by intracellular substrate concentrations. *S*_*int*_ can regulate either (A) transporter synthesis (*α*_*T*_) or (B) transporter downregulation (*γ*_*T*_). In both cases, we assume this regulation takes the form of a Hill equation with a 'saturation' constant of (A) *K*_*syn*_ or (B) *K*_*deg*_. The saturation constant is the equivalent to the standard disassociation constant but represents the concentration where the system is half saturated for its regulatory potential. For (A) and (B) the diagram on the right uses a hill coefficient (*n*) of 3, (case of *n* = 1 is in S1 Appendix, section VII part b, S1 Fig). Higher *n* leads to more robustness, but global perfect homeostasis is not achievable by any finite value of *n*. For both (A) and (B), the saturation constants are normalized by $S_{0}\;=\;\frac{k_{act}\alpha_{T}}{k_{\gamma_{S}}k_{\gamma_{T}}^{c}}$.

Above we described how activity-dependent downregulation can achieve global perfect homeostasis. This system is distinct from transcriptional regulation of transporter synthesis by high gain feedback of internal nutrient concentrations (e.g. negative feedback with high cooperativity) which is considered to be a homeostatic mechanism [10]. In this system, it is typically assumed that the synthesis rate of the transporter is decreasing as a function of the internal nutrient concentration, e.g. $\alpha_{T}^{}(S_{ext},S_{int})=k_{\alpha_{T}}\left(\frac{K_{M}^{n}}{K_{M}^{n}+S_{int}^{n}}\right)$ and that the transporter downregulation rate is constant,$k_{\gamma_{T}}(S_{ext},S_{int})=\gamma_{T}$. It is easy to see that in this case the condition in Eq (4) is not met, i.e. $k_{\gamma_{T}}/\alpha_{T}$ does not depend on *S*_*ext*_, and thus this system *cannot* achieve global perfect homeostasis for any finite *n* (Fig 6; S1 Appendix, section VII).

**Fig 7:**
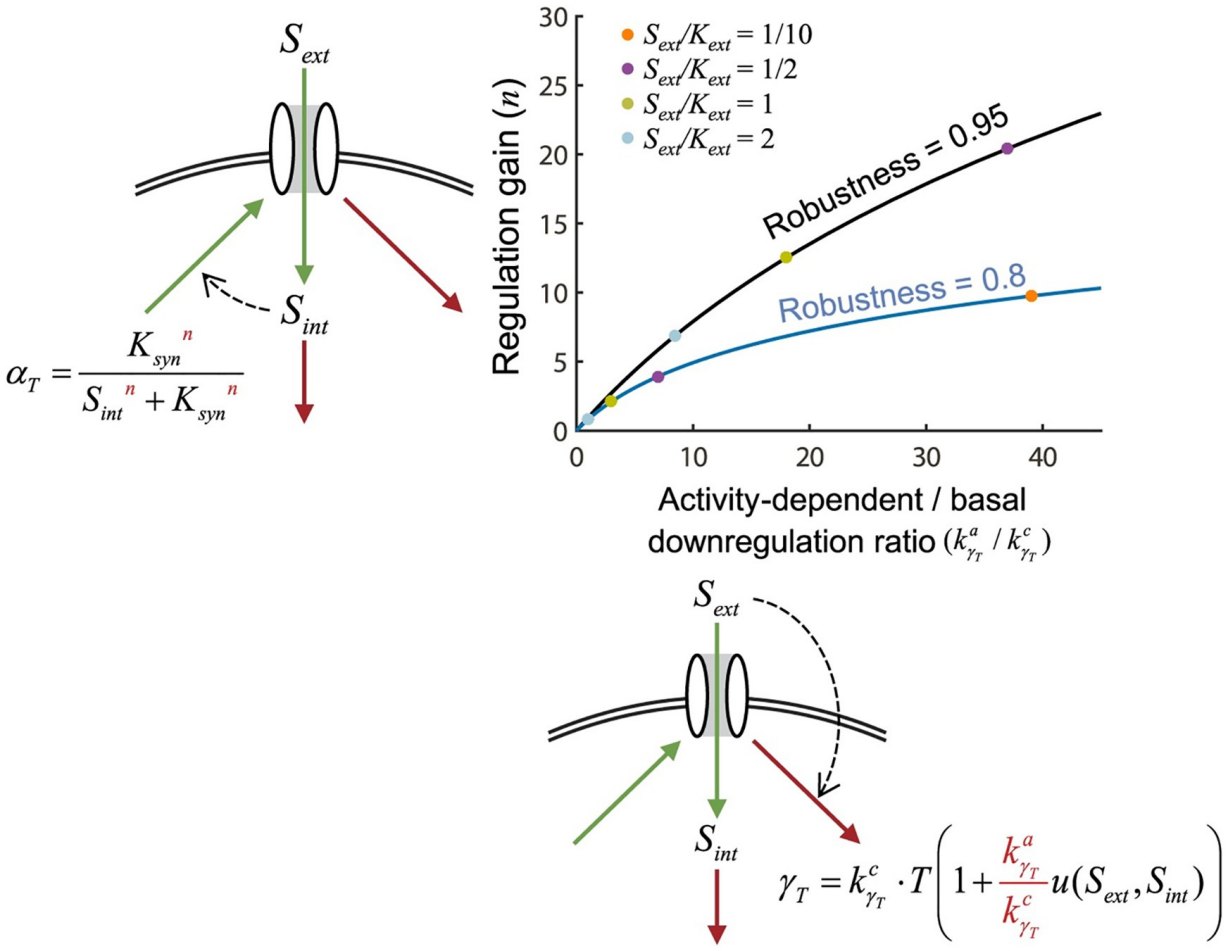
Comparison of internal nutrient-dependent regulation of transporter synthesis and activity-dependent transporter downregulation. For different normalized concentrations of *S*_*ext*_, the values of *n* and $k_{\gamma_{T}}^{a}/k_{\gamma_{T}}^{c}$ required to achieve either 0.8 or 0.95 robustness (blue curve and black curve respectively) are plotted. Four different specific normalized *S*_*ext*_ values are represented by the four colored dots. We assumed nutrient uptake per transporter was Michaelian $u(S_{ext})=\frac{S_{ext}}{S_{ext}+K_{ext}}$.

Under real biological conditions, neither internal nutrient sensing nor activity-dependent sensing can achieve global perfect homeostasis. The two regulatory systems can be compared based on the parameters required to achieve the same robustness, *r* (Fig 7). Internal nutrient sensing approaches global perfect homeostasis when *n* is large. But, high cooperativity is mechanistically difficult to achieve. Activity-dependent downregulation approaches global perfect homeostasis when $k_{\gamma_{T}}^{a}\;>>\;k_{\gamma_{T}}^{c}$. High levels of activity-dependent downregulation are easy to achieve mechanistically but might come at a high cost due to increased protein turnover.

**Fig 8:**
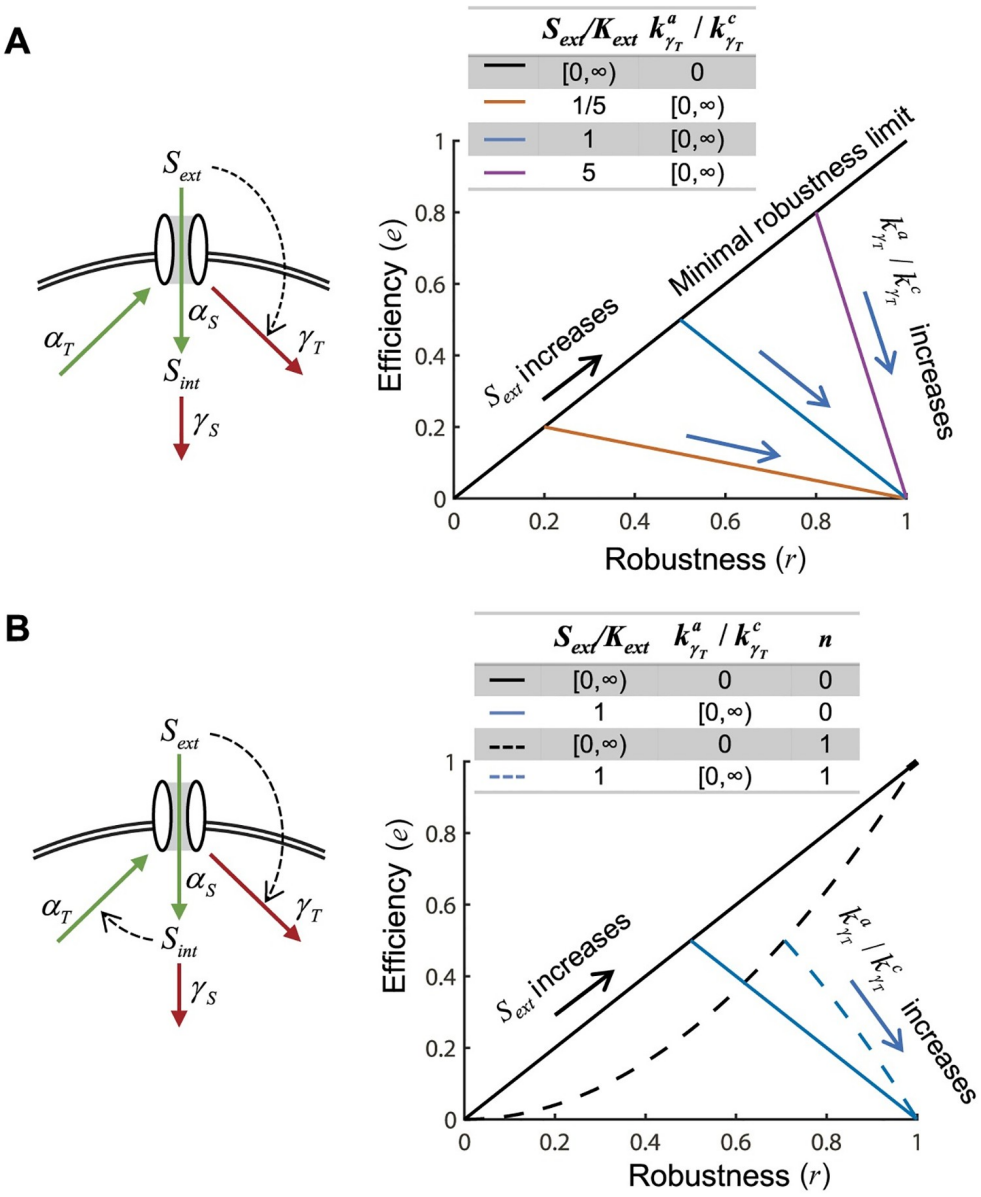
The trade-off between robustness and efficiency. **(A)** Efficiency is defined as the total nutrient uptake of a single transporter over its average lifetime. As activity-dependent downregulation increases (orange, blue and purple lines with direction of increase denoted by blue arrows), robustness increases and efficiency decreases. The minimal value of robustness depends on the *S*_*ext*_ (black line). Therefore, as *S*_*ext*_ increases (black arrow), the slope of the efficiency-robustness trade-off increases (orange versus blue versus purple lines). **(B)** Robustness-efficiency trade-off in a system with only activity-dependent downregulation (solid lines) or with activity-dependent downregulation and internal nutrient regulation of transporter synthesis (dashed lines). Adding internal nutrient dependent transporter regulation results in improving the minimal robustness limit and the robustness-efficiency trade-off. As *n* increases so does the improvement in the trade-off (S1 Appendix, section VIII, S2 Fig, Fig 7).

To explore the potential trade-off due to cost that comes with activity-dependent regulation, we used two metrics: efficiency and robustness. We defined efficiency as the total nutrient uptake of a single transporter over its average lifetime. Robustness is defined above. When there is no activity-dependent downregulation, robustness takes on its minimal value and efficiency its maximal value (Fig 8A; S1 Appendix, section VIII). As $k_{\gamma_{T}}^{a}/k_{\gamma_{T}}^{c}$ is increased, robustness increases but efficiency goes down.

As most nutrient homeostatic systems likely utilize both internal nutrient and activity-dependent regulation, we tested whether the combination improved the robustness-efficiency trade-off. We first asked whether the combined system is still able to achieve the global perfect homeostasis that activity-dependent downregulation is able to achieve alone. Indeed, the combined system still satisfies Eq (4) and thus can still achieve global perfect homeostasis (Fig 2). But this combined system might have additional desirable properties over a system with just activity-dependent regulation. Indeed, transcriptional repression by internal nutrient levels improves the efficiency of the system for any given robustness (Fig 8B). As *n* is increased, the minimal robustness of the system is also increased (Fig 8B). In addition to the minimal robustness increasing, the trade-off between robustness and efficiency also has a higher slope such that for the same efficiency, higher robustness is achieved and vice versa (Fig 8B; S1 Appendix, section VIII). This result is intuitive—when nutrient levels are high, instead of just degrading transporter, the combined systems allows fewer transporters to be made.

## Discussion

We analyzed a general nutrient uptake system and derived the conditions for global perfect homeostasis. For a large family of scenarios, the internal nutrient can be made independent of the external nutrient if the ratio of transporter synthesis and depletion rates explicitly depends on the net uptake rate of the nutrient from the environment. A simple way to achieve this regulation is for active transporters to be preferentially downregulated, that is, have activity-dependent downregulation.

While the transcriptional regulation of transporter gene synthesis has been relatively well characterized, the mechanisms that lead to the downregulation of plasma membrane transporters are less clear. Interestingly, a recent study of the yeast uracil transporter, Fur4p, suggests it exhibits properties that cause its degradation to depend on its activity. Specifically, there is evidence that binding of nutrients to the transporter causes it to adopt a conformation that marks it for ubiquitinylation, followed by endocytosis and degradation [52]. While this detailed analysis has not been performed on many transporters, it’s known that many transporters are endocytosed when nutrient levels increase. While previous work had suggested that this likely serves to protect cells from toxicity during acute increases in nutrient levels [39], we propose that this mechanism could also play a crucial role in homeostasis at all external nutrient concentrations.

Homeostasis can be regulated by internal and external nutrient concentrations. Both forms of regulation have limitations that affect their utility over all external concentrations, but both are useful in a range of biologically relevant regimes. Systems that integrate both internal and external nutrient concentrations can be robust over a wider range of concentrations (with lower total energy input) than systems that sense only internal or only external nutrient concentration. This complementarity may explain why these two modes of regulation are common in homeostatic systems.

While out of the scope of this work, in future work it will be interesting to expand on this simple system in several ways. While here we focused on steady-state behaviors, kinetic differences between different modes of regulation are likely important. For example, different mechanisms of action can accentuate kinetic difference, e.g. transcriptional versus post-translation control. In addition, many homeostatic systems utilize internal nutrient storage or nutrient recycling which in principal can affect the homeostatic response. Furthermore, analysis of addition constraints that can be placed on Eq (1) could identify other biologically achievable systems that satisfy the homeostatic relationship in Eq (2).

The results we derived here are relevant for any multi-compartment biological system that implements homeostasis, where there is flux among the compartments. This work thus should be useful as a guide in studying homeostasis in any biological system and well as in the design of synthetic ones.

## Methods and models

Detailed derivations can be found in S1 Appendix.

To determine the requirements for homeostasis for the general uptake system described in Eq (1), we used the method developed in [14]. In brief, we required the rule

rank(P|I)=rank(I) (7)

where *P* is a column of elements $p_{i}=\frac{\partial \text{log}(\sigma_{i})}{\partial \text{log}(S_{ext})}$ with *σ*_*i*_ = *α*_*S*_, *γ*_*S*_, *α*_*T*_, *γ*_*T*_, which are the uptake and usage fluxes of the internal nutrient and uptake and usage fluxes for transporter respectively; each of those could be a function of *S*_*ext*_, *S*_*int*_, and *T*. *I* is the matrix representation of largest parameter-independent subspace spanned by the columns of (*M* | *K*) where *M* is a column of elements $m_{i}=\frac{\partial \text{log}(\sigma_{i})}{\partial \text{log}(T)}$ with *σ*_*i*_ = *α*_*S*_, *γ*_*S*_, *α*_*T*_, *γ*_*T*_, and $K=\left(\begin{array}{cc} \alpha_{S}^{0} & 0\\ \gamma_{S}^{0} & 0\\ 0 & \alpha_{T}^{0}\\ 0 & \gamma_{T}^{0} \end{array}\right)$ where $\alpha_{S}^{0},\gamma_{S}^{0},\alpha_{T}^{0},\gamma_{T}^{0}$ are the steady state solutions of fluxes.

We constructed *P*|*I* and *I* matrixes and analytically found all the conditions that satisfy Eq (7). The homeostasis condition was analytically derived using the described above criterion. Criterion necessary for global perfect homeostasis for special cases of Eq (1) were analytically derived by substituting into the resulting general solution with simplified forms of *α*_*S*_, *γ*_*S*_, *α*_*T*_, *γ*_*T*_.

We considered several simplified conditions. If transporter production is independent of transporter concentration the following homeostasis condition was obtained

$$\frac{\gamma_{T}(S_{ext},S_{int},T)}{\alpha_{T}^{}(S_{ext},S_{int})}\propto \frac{\alpha_{S}^{}(S_{ext},S_{int},T)}{\gamma_{S}(S_{ext},S_{int})}\cdot func(S_{int}).$$ (8)

where *func*(*S*_*int*_) is any function of internal nutrient concentration.

To evaluate homeostasis we used two metrics: efficiency: *e* = *α*_*S*_ / *γ*_*T*_ - the total nutrient uptake of a single transporter over its average lifetime and robustness: *r* = *S*_*int*_ / *S*_*int*,*max*_ where *S*_*int*,*max*_ is the maximum *S*_*int*_ across all *S*_*ext*_.

To compare activity-dependent transporter downregulation with the internal nutrient based transcriptional regulation we compared the following two systems:

$$\begin{array}{ll} {\dot{S}}_{int} & =k_{cat}\cdot T\cdot \frac{S_{ext}}{S_{ext}+K_{ext}}-k_{\gamma_{S}}\cdot S_{int}\\ \dot{T} & =\alpha_{T}\frac{K_{syn}{}^{n}}{K_{syn}{}^{n}+S_{int}{}^{n}}-k_{\gamma_{T}}^{c}T \end{array}$$ (9)

and

$$\begin{array}{ll} {\dot{S}}_{int} & =k_{cat}\cdot T\cdot \frac{S_{ext}}{S_{ext}+K_{ext}}-k_{\gamma_{S}}\cdot S_{int}\\ \dot{T} & =\alpha_{T}-k_{\gamma_{T}}^{}\frac{S_{int}{}^{n}}{K_{\text{deg}}{}^{n}+S_{int}{}^{n}}\cdot T \end{array}$$ (10)

where *K*_*syn*_ and *K*_*deg*_ are saturation constants and *n* is the Hill coefficient.

To compare a system with internal nutrient based regulation of synthesis to a system with activity-dependent transporter downregulation we analytically calculated the parameters required to achieve a threshold robustness of *r* = 0.95 and *r* = 0.8 (Fig 7). In addition, we analytically compared the efficiency-robustness dependence in a system with both internal nutrient based regulation of synthesis and activity-dependent transporter downregulation (*n* = 0, 1 in Fig 8; *n* = 0, 1, 3, 10 in S2 Fig).

All analytical solutions were found in Mathematica version 10.2 (Wolfram Research) and were visualized in MATLAB R2015a (MathWorks).

## Supporting information

S1 Appendix Detailed mathematical derivations of the equations presented in the main text. (DOCX)

S1 Fig Michaelian regulation by intracellular nutrient concentrations. Robustness for a homeostatic system with Michaelian regulation by internal nutrient concentrations (*n* = 1) (**A**) transcriptional repression by *S*_*int*_ with a saturation constant of *K*_*syn*_ or (**B**) transporter downregulation by *S*_*int*_ a with saturation constant of *K*_*deg*_.
(TIF)

S2 FigThe trade-off between robustness and efficiency with high order regulation.Robustness-efficiency trade-off with different orders of transporter synthesis regulation (different line style for different value of *n*). A system without activity-dependent transporter downregulation for a range of *S*_*ext*_ concentrations (black). Different ratios between activity-dependent transporter downregulation and basal degradation $k_{\gamma_{T}}^{a}/k_{\gamma_{T}}^{c}$ with constant external concentration (*S*_*ext*_ = *K*_*ext*_) (blue).(TIF)

## References

[1] Langley LL. Homeostasis: origins of the concept. Dowden Hutchinson and Ross; 1973.

[2] Schneck DJ. Feedback control and the concept of homeostasis. Mathematical Modelling. 1987.

[3] Eide DJ. Multiple regulatory mechanisms maintain zinc homeostasis in Saccharomyces cerevisiae. J Nutr. 2003rd ed. 2003;133: 1532S–5S. 10.1093/jn/133.5.1532S 12730459

[4] Walker JD, Enache M, Dearden JC. Quantitative cationic-activity relationships for predicting toxicity of metals. Environmental toxicology and chemistry / SETAC. 2003rd ed. 2003;22: 1916–1935. 10.1897/02-568 12924590

[5] Vander Heiden MG, Cantley LC, Thompson CB. Understanding the Warburg Effect: The Metabolic Requirements of Cell Proliferation. Science. 2009;324: 1029–1033. 10.1126/science.1160809 19460998 PMC2849637

[6] DeBerardinis RJ, Lum JJ, Hatzivassiliou G, Thompson CB. The biology of cancer: metabolic reprogramming fuels cell growth and proliferation. Cell Metabolism. 2008 ed. 2008;7: 11–20. 10.1016/j.cmet.2007.10.002 18177721

[7] Levine AJ, Puzio-Kuter AM. The control of the metabolic switch in cancers by oncogenes and tumor suppressor genes. Science. 2010 ed. 2010;330: 1340–1344. 10.1126/science.1193494 21127244

[8] Marshall S. Role of insulin, adipocyte hormones, and nutrient-sensing pathways in regulating fuel metabolism and energy homeostasis: a nutritional perspective of diabetes, obesity, and cancer. Sci STKE. 2006 ed. 2006;2006: re7. 10.1126/stke.3462006re7 16885148

[9] Locasale JW, Cantley LC, Vander Heiden MG. Cancer's insatiable appetite. Nat Biotechnol. 2009 ed. 2009;27: 916–917. 10.1038/nbt1009-916 PMC3744822 19816448

[10] Khammash M. An engineering viewpoint on biological robustness. BMC Biology. BMC Biology; 2016;: 1–11. 27007299 10.1186/s12915-016-0241-x PMC4804522

[11] Eide DJ. The molecular biology of metal ion transport in Saccharomyces cerevisiae. Annual review of nutrition. 1998 ed. 1998;18: 441–469. 10.1146/annurev.nutr.18.1.441 9706232

[12] Shinar G, Feinberg M. Structural sources of robustness in biochemical reaction networks. Science. 2010;327: 1389–1391. 10.1126/science.1183372 20223989

[13] Stelling J, Sauer U, Szallasi Z, Doyle FJ, Doyle J. Robustness of cellular functions. Cell. 2004;118: 675–685. 10.1016/j.cell.2004.09.008 15369668

[14] Steuer R, Waldherr S, Sourjik V, Kollmann M. Robust signal processing in living cells. PLoS Comput Biol. 2011;7: e1002218 10.1371/journal.pcbi.1002218 22215991 PMC3219616

[15] Heredia J, Crooks M, Zhu Z. Phosphorylation and Cu+ coordination-dependent DNA binding of the transcription factor Mac1p in the regulation of copper transport. J Biol Chem. 2001;276: 8793–8797. 10.1074/jbc.M008179200 11134042

[16] Philpott CC, Protchenko O. Response to iron deprivation in Saccharomyces cerevisiae. Eukaryotic Cell. 2008;7: 20–27. 10.1128/EC.00354-07 17993568 PMC2224162

[17] Yamaguchi-Iwai Y, Serpe M, Haile D, Yang W, Kosman DJ, Klausner RD, et al Homeostatic regulation of copper uptake in yeast via direct binding of MAC1 protein to upstream regulatory sequences of FRE1 and CTR1. J Biol Chem. 1997;272: 17711–17718. 9211922 10.1074/jbc.272.28.17711

[18] Zhao H, Butler E, Rodgers J, Spizzo T, Duesterhoeft S, Eide D. Regulation of zinc homeostasis in yeast by binding of the ZAP1 transcriptional activator to zinc-responsive promoter elements. J Biol Chem. 1998;273: 28713–28720. 9786867 10.1074/jbc.273.44.28713

[19] Ozcan S, Johnston M. Function and regulation of yeast hexose transporters. Microbiology and Molecular Biology Reviews. 1999 ed. 1999;63: 554–569. 10.1128/mmbr.63.3.554-569.1999 PMC103746 10477308

[20] Yano K, Fukasawa T. Galactose-dependent reversible interaction of Gal3p with Gal80p in the induction pathway of Gal4p-activated genes of Saccharomyces cerevisiae. Proc Natl Acad Sci USA. 1997;94: 1721–1726. 9050845 10.1073/pnas.94.5.1721 PMC19983

[21] Oshima Y. The phosphatase system in Saccharomyces cerevisiae. Genes Genet Syst. 1997;72: 323–334. 9544531 10.1266/ggs.72.323

[22] Vander Heiden MG, Cantley LC, Thompson CB. Understanding the Warburg Effect: The Metabolic Requirements of Cell Proliferation. Science. 2009;324: 1029–1033. 10.1126/science.1160809 19460998 PMC2849637

[23] Kulkarni AA, Abul-Hamd AT, Rai R, Berry El H, Cooper TG. Gln3p nuclear localization and interaction with Ure2p in Saccharomyces cerevisiae. J Biol Chem. 2001;276: 32136–32144. 10.1074/jbc.M104580200 11408486 PMC4384441

[24] Stanbrough M, Magasanik B. Two transcription factors, Gln3p and Nil1p, use the same GATAAG sites to activate the expression of GAP1 of Saccharomyces cerevisiae. Journal of Bacteriology. 1996;178: 2465–2468. 8636059 10.1128/jb.178.8.2465-2468.1996 PMC177966

[25] Hicke L. Ubiquitin-dependent internalization and down-regulation of plasma membrane proteins. FASEB J. 1997;11: 1215–1226. 9409540 10.1096/fasebj.11.14.9409540

[26] Petris MJ, Mercer JF, Culvenor JG, Lockhart P, Gleeson PA, Camakaris J. Ligand-regulated transport of the Menkes copper P-type ATPase efflux pump from the Golgi apparatus to the plasma membrane: a novel mechanism of regulated trafficking. The EMBO Journal. 1996;15: 6084–6095. 8947031 PMC452430

[27] Frey AG, Bird AJ, Evans-Galea MV, Blankman E, Winge DR, Eide DJ. Zinc-regulated DNA binding of the yeast Zap1 zinc-responsive activator. PLoS One. 2011;6: e22535 10.1371/journal.pone.0022535 21799889 PMC3142189

[28] Gitan RS, Luo H, Rodgers J, Broderius M, Eide D. Zinc-induced inactivation of the yeast ZRT1 zinc transporter occurs through endocytosis and vacuolar degradation. J Biol Chem. 1998 ed. 1998;273: 28617–28624. 10.1074/jbc.273.44.28617 9786854

[29] Graschopf A, Stadler JA, Hoellerer MK, Eder S, Sieghardt M, Kohlwein SD, et al The yeast plasma membrane protein Alr1 controls Mg2+ homeostasis and is subject to Mg2+-dependent control of its synthesis and degradation. J Biol Chem. 2001;276: 16216–16222. 10.1074/jbc.M101504200 11279208

[30] Lai K, McGraw P. Dual control of inositol transport in Saccharomyces cerevisiae by irreversible inactivation of permease and regulation of permease synthesis by INO2, INO4, and OPI1. J Biol Chem. 1994 ed. 1994;269: 2245–2251. 8294482

[31] Lau WT, Howson RW, Malkus P, Schekman R, O'Shea EK. Pho86p, an endoplasmic reticulum (ER) resident protein in Saccharomyces cerevisiae, is required for ER exit of the high-affinity phosphate transporter Pho84p. Proc Natl Acad Sci USA. 2000;97: 1107–1112. 10655492 10.1073/pnas.97.3.1107 PMC15537

[32] Ooi CE, Rabinovich E, Dancis A, Bonifacino JS, Klausner RD. Copper-dependent degradation of the Saccharomyces cerevisiae plasma membrane copper transporter Ctr1p in the apparent absence of endocytosis. The EMBO Journal. 1996 ed. 1996;15: 3515–3523. PMC451948 8670854

[33] Felice MR, De Domenico I, Li L, Ward DM, Bartok B, Musci G, et al Post-transcriptional regulation of the yeast high affinity iron transport system. J Biol Chem. 2005;280: 22181–22190. 10.1074/jbc.M414663200 15817488

[34] Gitan RS, Eide DJ. Zinc-regulated ubiquitin conjugation signals endocytosis of the yeast ZRT1 zinc transporter. Biochem J. 2000;346 Pt 2: 329–336. 10677350 PMC1220857

[35] Liu J, Sitaram A, Burd CG. Regulation of copper-dependent endocytosis and vacuolar degradation of the yeast copper transporter, Ctr1p, by the Rsp5 ubiquitin ligase. Traffic. 2007;8: 1375–1384. 10.1111/j.1600-0854.2007.00616.x 17645432

[36] Horak J, Wolf DH. Glucose-Induced Monoubiquitination of the Saccharomyces cerevisiae Galactose Transporter Is Sufficient To Signal Its Internalization. Journal of Bacteriology. 2001;183: 3083–3088. 10.1128/JB.183.10.3083-3088.2001 11325936 PMC95208

[37] Lundh F, Mouillon JM, Samyn D, Stadler K, Popova Y, Lagerstedt JO, et al Molecular mechanisms controlling phosphate-induced downregulation of the yeast Pho84 phosphate transporter. Biochemistry. 2009;48: 4497–4505. 10.1021/bi9001198 19348508

[38] Hein C, Springael JY, Volland C, Haguenauer-Tsapis R, Andre B. NPl1, an essential yeast gene involved in induced degradation of Gap1 and Fur4 permeases, encodes the Rsp5 ubiquitin-protein ligase. Mol Microbiol. 1995;18: 77–87. 8596462 10.1111/j.1365-2958.1995.mmi_18010077.x

[39] Eide DJ. An “Inordinate Fondness for Transporters” Explained? Sci Signal. 2012;5: pe5–. 10.1126/scisignal.2002837 22317920

[40] Hicke L, Dunn R. Regulation of membrane protein transport by ubiquitin and ubiquitin-binding proteins. Annu Rev Cell Dev Biol. 2003rd ed. 2003;19: 141–172. 10.1146/annurev.cellbio.19.110701.154617 14570567

[41] Lauwers E, Erpapazoglou Z, Haguenauer-Tsapis R, Andre B. The ubiquitin code of yeast permease trafficking. Trends Cell Biol. 2010 ed. 2010;20: 196–204. 10.1016/j.tcb.2010.01.004 20138522

[42] Leon S, Haguenauer-Tsapis R. Ubiquitin ligase adaptors: regulators of ubiquitylation and endocytosis of plasma membrane proteins. Experimental Cell Research. 2008 ed. 2009;315: 1574–1583. 10.1016/j.yexcr.2008.11.014 19070615

[43] Kotte O, Zaugg JB, Heinemann M. Bacterial adaptation through distributed sensing of metabolic fluxes. Mol Syst Biol. 2010;6. 10.1038/msb.2010.10 PMC2858440 20212527

[44] Kochanowski K, Volkmer B, Gerosa L, van Rijsewijk BRH, Schmidt A, Heinemann M. Functioning of a metabolic flux sensor in Escherichia coli. Proceedings of the …. 2013. 10.1073/pnas.1202582110 PMC3549114 23277571

[45] Drengstig T, Jolma IW, Ni XY, Thorsen K, Xu XM, Ruoff P. A Basic Set of Homeostatic Controller Motifs. Biophysj. Biophysical Society; 2012;103: 2000–2010. 10.1016/j.bpj.2012.09.033 PMC3491718 23199928

[46] Drengstig T, Ni XY, Thorsen K, Jolma IW, Ruoff P. Robust Adaptation and Homeostasis by Autocatalysis. J Phys Chem B. 2012;116: 5355–5363. 10.1021/jp3004568 22506960

[47] Ni XY, Drengstig T, Ruoff P. The Control of the Controller: Molecular Mechanisms for Robust Perfect Adaptation and Temperature Compensation. Biophys J. 2009;97: 1244–1253. 10.1016/j.bpj.2009.06.030 19720012 PMC2749762

[48] Drengstig T, Kjosmoen T, Ruoff P. Studying Adaptation and Homeostatic Behaviors of Kinetic Networks by Using MATLAB Methods in Molecular Biology. Totowa, NJ: Humana Press; 2011 pp. 153–172. 10.1007/978-1-61779-086-7_8 21468989

[49] Fersht A. Enzyme structure and function. WH Freeman & Co, New York Friedrich CG & Mitrenga G (1981) Oxidation of thiosulfate byParacoccus denitrificans and other hydrogen bacteria FEMS Microbiol Lett. WH Freeman & Co; 1985;10: 209–212.

[50] Fersht A. Structure and mechanism in protein science: a guide to enzyme catalysis and protein folding (book) Freeman. New York New York; 1998.

[51] Panikov NS. Kinetics, Microbial Growth Encyclopedia of Bioprocess Technology. John Wiley & Sons, Inc; 2002.

[52] Keener JM, Babst M. Quality control and substrate-dependent downregulation of the nutrient transporter Fur4. Traffic. 2013;14: 412–427. 10.1111/tra.12039 23305501 PMC3594327

[53] Ibáñez I, Díez-Guerra FJ, Giménez C, Zafra F. Neuropharmacology. Neuropharmacology. Elsevier Ltd; 2016;107: 376–386. 10.1016/j.neuropharm.2016.03.042 27044663

[54] Weyand S, Shimamura T, Yajima S, Suzuki S, Mirza O, Krusong K, et al Structure and Molecular Mechanism of a Nucleobase-Cation-Symport-1 Family Transporter. Science. American Association for the Advancement of Science; 2008;322: 709–713. 10.1126/science.1164440 PMC2885439 18927357

[55] Shimamura T, Weyand S, Beckstein O, Rutherford NG, Hadden JM, Sharples D, et al Molecular Basis of Alternating Access Membrane Transport by the Sodium-Hydantoin Transporter Mhp1. Science. 2010;328: 470–473. 10.1126/science.1186303 20413494 PMC2885435

[56] Lefkowitz RJ. G protein-coupled receptor kinases. Cell. 1993;74: 409–412. 8394218 10.1016/0092-8674(93)80042-d

[57] Krupnick JG, Benovic JL. The role of receptor kinases and arrestins in G protein-coupled receptor regulation. Annu Rev Pharmacol Toxicol. 1998;38: 289–319. 10.1146/annurev.pharmtox.38.1.289 9597157

[58] Ferguson SS. Evolving concepts in G protein-coupled receptor endocytosis: the role in receptor desensitization and signaling. Pharmacol Rev. 2001;53: 1–24. 11171937

[59] Ferguson SS, Caron MG. G protein-coupled receptor adaptation mechanisms. Semin Cell Dev Biol. 1998;9: 119–127. 10.1006/scdb.1997.0216 9599406

[60] Ferguson S, Barak LS, Zhang J. G-protein-coupled receptor regulation: role of G-protein-coupled receptor kinases and arrestins. Canadian journal of …. 1996. 10.1139/cjpp-74-10-1095 9022829

[61] Levoye A, Zwier JM, Jaracz-Ros A, Klipfel L, Cottet M, Maurel D, et al A Broad G Protein-Coupled Receptor Internalization Assay that Combines SNAP-Tag Labeling, Diffusion-Enhanced Resonance Energy Transfer, and a Highly Emissive Terbium Cryptate. Front Endocrinol. 2015;6: 1723. 10.3389/fendo.2015.00167 PMC4638144 26617570

[62] Graham GJ, Locati M, Mantovani A, Rot A, Thelen M. Immunology Letters. Immunology Letters. Elsevier B.V; 2012;145: 30–38. 10.1016/j.imlet.2012.04.004 22698181

[63] Russell CB, Stewart RC, Dahlquist FW. Control of transducer methylation levels in Escherichia coli: investigation of components essential for modulation of methylation and demethylation reactions. Journal of Bacteriology. 1989;171: 3609–3618. 2661528 10.1128/jb.171.7.3609-3618.1989 PMC210102

[64] Barkai N, Leibler S. Robustness in simple biochemical networks. Nature. 1997;387: 913–917. 10.1038/43199 9202124

[65] Alon U, Surette MG, Barkai N, Leibler S. Robustness in bacterial chemotaxis. Nature. 1999;397: 168–171. 10.1038/16483 9923680

[66] Popova Y, Thayumanavan P, Lonati E, Agrochão M, Thevelein JM. Transport and signaling through the phosphate-binding site of the yeast Pho84 phosphate transceptor. Proceedings of the National Academy of Sciences. 2010;107: 2890–2895. 10.1073/pnas.0906546107 PMC2840322 20133652

[67] Ozcan S. Glucose sensing and signaling by two glucose receptors in the yeast Saccharomyces cerevisiae. The EMBO Journal. 1998;17: 2566–2573. 10.1093/emboj/17.9.2566 9564039 PMC1170598

[68] Iraqui I, Vissers S, Bernard F, de Craene J-O, Boles E, Urrestarazu A, et al Amino Acid Signaling in Saccharomyces cerevisiae: a Permease-Like Sensor of External Amino Acids and F-Box Protein Grr1p Are Required for Transcriptional Induction of the AGP1Gene, Which Encodes a Broad-Specificity Amino Acid Permease. Mol Cell Biol. 1999;19: 989–1001. 9891035 10.1128/mcb.19.2.989 PMC116030

[69] Díez-Sampedro A, Hirayama BA. A glucose sensor hiding in a family of transporters. … National Academy …. 2003. 10.1073/pnas.1733027100 PMC208830 13130073

[70] Didion T, Regenberg B, Jørgensen MU, Kielland-Brandt MC, Andersen HA. The permease homologue Ssy1p controls the expression of amino acid and peptide transporter genes in Saccharomyces cerevisiae. Mol Microbiol. Blackwell Science Ltd, UK; 1998;27: 643–650. 10.1046/j.1365-2958.1998.00714.x 9489675

[71] Schwöppe C, Winkler HH, Neuhaus HE. Connection of transport and sensing by UhpC, the sensor for external glucose-6-phosphate in Escherichia coli. The FEBS Journal. Blackwell Science Ltd; 2003;270: 1450–1457. 10.1046/j.1432-1033.2003.03507.x 12654000

[72] Biswas K, Morschhäuser J. The Mep2p ammonium permease controls nitrogen starvation-induced filamentous growth in Candida albicans. Mol Microbiol. Blackwell Science Ltd; 2005;56: 649–669. 10.1111/j.1365-2958.2005.04576.x 15819622

[73] Van Nuland A, Vandormael P, Donaton M, Alenquer M, Lourenço A, Quintino E, et al Ammonium permease-based sensing mechanism for rapid ammonium activation of the protein kinase A pathway in yeast. Mol Microbiol. Blackwell Publishing Ltd; 2006;59: 1485–1505. 10.1111/j.1365-2958.2005.05043.x 16468990

[74] Donaton MCV, Holsbeeks I, Lagatie O, Van Zeebroeck G, Crauwels M, Winderickx J, et al The Gap1 general amino acid permease acts as an amino acid sensor for activation of protein kinase A targets in the yeast Saccharomyces cerevisiae. Mol Microbiol. Blackwell Publishing Ltd; 2003;50: 911–929. 10.1046/j.1365-2958.2003.03732.x 14617151

[75] Giots F, Donaton MCV, Thevelein JM. Inorganic phosphate is sensed by specific phosphate carriers and acts in concert with glucose as a nutrient signal for activation of the protein kinase A pathway in the yeast Saccharomyces cerevisiae. Mol Microbiol. Blackwell Science Ltd; 2003;47: 1163–1181. 10.1046/j.1365-2958.2003.03365.x 12581367

[76] Walch Liu P, Forde BG. Nitrate signalling mediated by the NRT1.1 nitrate transporter antagonises l-glutamate-induced changes in root architecture. The Plant Journal. Blackwell Publishing Ltd; 2008;54: 820–828. 10.1111/j.1365-313X.2008.03443.x 18266918

[77] Hyde R, Cwiklinski EL, MacAulay K, Taylor PM, Hundal HS. Distinct Sensor Pathways in the Hierarchical Control of SNAT2, a Putative Amino Acid Transceptor, by Amino Acid Availability. Journal of Biological Chemistry. 2007;282: 19788–19798. 10.1074/jbc.M611520200 17488712

[78] Goberdhan DCI. PAT-related amino acid transporters regulate growth via a novel mechanism that does not require bulk transport of amino acids. Development. 2005;132: 2365–2375. 10.1242/dev.01821 15843412

[79] Bianchi L, Díez-Sampedro A. A Single Amino Acid Change Converts the Sugar Sensor SGLT3 into a Sugar Transporter. Feany MB, editor. PLoS One. 2010;5: e10241 10.1371/journal.pone.0010241 20421923 PMC2857651

[80] Schneider YJ. Fate of plasma membrane during endocytosis. I. Uptake and processing of anti-plasma membrane and control immunoglobulins by cultured fibroblasts. J Cell Biol. 1979;82: 449–465. 479309 10.1083/jcb.82.2.449 PMC2110459

[81] Schneider Y. Fate of plasma membrane during endocytosis. II. Evidence for recycling (shuttle) of plasma membrane constituents. J Cell Biol. 1979;82: 466–474. 479310 10.1083/jcb.82.2.466 PMC2110464

[82] Wileman T, Harding C, Stahl P. Receptor-mediated endocytosis. Biochem J. 1985;232: 1–14. 2867759 10.1042/bj2320001 PMC1152830

[83] Clifford RJ, Maryon EB, Kaplan JH. Dynamic internalization and recycling of a metal ion transporter: Cu homeostasis and CTR1, the human Cu+ uptake system. J Cell Sci. The Company of Biologists Ltd; 2016;129: 1711–1721. 10.1242/jcs.173351 PMC4852764 26945057

[84] Guerinot ML. The ZIP family of metal transporters. Biochimica et Biophysica Acta (BBA)—Biomembranes. 2000. 10.1016/s0005-2736(00)00138-3 10748254

