# Supplementary material for: Achieving global perfect homeostasis through transporter regulation
Source: PLoS Comput Biol. 2017 Apr 17;13(4):e1005458. doi: 10.1371/journal.pcbi.1005458 (PMC5411106; doi:10.1371/journal.pcbi.1005458)
Supplement: S1 Appendix — (DOCX) [file pcbi.1005458.s001.docx]

**Supplementary information**

[**I.** **The general system** 2](#_Toc478423224)

[**II.** **Nutrient uptake is proportional to the number of transporters** 5](#_Toc478423225)

[**III.** **Michaelian uptake** 6](#_Toc478423226)

[**IV.** **Mechanism of activity-dependent downregulation** 7](#_Toc478423227)

[**V.** **Analyzing deviations from global perfect homeostasis** 7](#_Toc478423228)

[**VI.** **Regulation by an independent sensor that senses external nutrient concentration** 9](#_Toc478423229)

[**VII.** **Regulation by the internal nutrient concentrations** 10](#_Toc478423230)

[**a.** **Transporter expression that depends on the internal nutrient concentration** 10](#_Toc478423231)

[**b.** **Transporters downregulation that depends on the internal nutrient concentration** 12](#_Toc478423232)

[**VIII.** **Trade-off between robustness and efficiency** 13](#_Toc478423233)

1. **The general system**

We analyzed a general homeostatic system using the framework presented by Steuer et. al. [1]. We assume the following dynamic system composed of external nutrient,$S_{ext}$, an internal nutrient, $S_{int}$, and a membrane transporter, $T$,

where $\alpha_{S}(T,S_{int},S_{ext})$ and $\gamma_{S}(S_{int},S_{ext})$are nutrient uptake and usage fluxes respectively; $\alpha_{T}(T,S_{int},S_{ext})$ and $\gamma_{T}(T,S_{int},S_{ext})$are transporter synthesis and downregulation fluxes respectively.

In matrix form,

We assumed the steady state solution exists, not necessarily unique, such that

$$N\cdot v^{0}=0$$

where *ν^0^* is the reaction fluxes steady state vector.

The matrix *K* is defined as,

In our case,

$$K=\left(\begin{matrix} \alpha_{S}^{0} & 0 \\ \gamma_{S}^{0} & 0 \\ 0 & \alpha_{T}^{0} \\ 0 & \gamma_{T}^{0} \end{matrix}\right)$$

where $\alpha_{S}^{0},\gamma_{S}^{0},\alpha_{T}^{0},\gamma_{T}^{0}$ denote the stationary flux values.

The “perturbation vector” is given by the logarithmic derivative of the fluxes with respect to the changing variable. In our case, the perturbed variable is *S_ext_*,

$$P=\left(\begin{matrix} p_{1} \\ p_{2} \\ p_{3} \\ p_{4} \end{matrix}\right)$$

where $p_{i}=\frac{\partial\log(\sigma_{i})}{\partial\log(S_{ext})}$ with$\sigma_{i}=\alpha_{S},\gamma_{S},\alpha_{T},\gamma_{T}$.

The vector *M* is defined as the logarithmic derivative of the fluxes with respect to the controlled variable. In our case, the controlled variable is $T$, thus,

$$M=\left(\begin{matrix} m_{1} \\ 0 \\ m_{3} \\ m_{4} \end{matrix}\right)$$

where $m_{i}=\frac{\partial\log(\sigma_{i})}{\partial\log(T)}$ with$\sigma_{i}=\alpha_{S},\gamma_{S},\alpha_{T},\gamma_{T}$.

For *local perfect* homeostasis (robustness to perturbation around a steady state solution)

$\text{rank}(P|M|K)=\text{rank}(M|K)$ .

Note that this condition *depends* on the steady state solution.

By *global perfect* homeostasis\ the concentration of the robust variable is invariant with respect to changes in the perturbed variable. The condition for *global* perfect robustness is

$$\text{rank}(P|I)=\mathrm{rank}(I)$$

where *I* is the matrix whose columns are spanned by the largest vector space that allows local robustness.

In our case, the reduced form of *I* is

$$I=\left(\begin{matrix} 1 & 1 & 0 \\ 0 & 1 & 0 \\ m_{3}/m_{1} & 0 & 1 \\ m_{4}/m_{1} & 0 & 1 \end{matrix}\right)$$

and thus the rank(*I*)=3. (*P*|*I*) reduces to,

$$(P|I)=\left(\begin{matrix} 1 \\ p_{2}/p_{1} \\ p_{3}/p_{1} \\ p_{4}/p_{1} \end{matrix}\begin{matrix} 1 & 1 & 0 \\ 0 & 1 & 0 \\ m_{3}/m_{1} & 0 & 1 \\ m_{4}/m_{1} & 0 & 1 \end{matrix}\right)\to\left(\begin{matrix} 1 \\ p_{2}/p_{1} \\ 0 \\ \frac{p_{4}-p_{3}}{p_{1}} \end{matrix}\begin{matrix} 1 & 1 & 0 \\ 0 & 1 & 0 \\ 0 & 0 & 1 \\ \frac{m_{4}-m_{3}}{m_{1}} & 0 & 1 \end{matrix}\right)$$

It’s clear that the rank of (*P|I*) = 3 only if,

$$\frac{p_{4}-p_{3}}{p_{1}}=\frac{m_{4}-m_{3}}{m_{1}}(1-p_{2}/p_{1})$$

that is,

$$\frac{\partial_{\log(S_{ext})}\log(\gamma_{T}/\alpha_{T})}{\partial_{\log(S_{ext})}\log(\alpha_{S})}=\frac{\partial_{\log(\mathrm{T})}\log(\gamma_{T}/\alpha_{T})}{\partial_{\log(\mathrm{T})}\log(\alpha_{S})}(1-\frac{\partial_{\log(S_{ext})}\log(\gamma_{S})}{\partial_{\log(S_{ext})}\log(\alpha_{S})})$$

Using $\partial_{\log x}\log y=\frac{x}{y}\partial_{x}y$, Eq. can be reduced to the following form:

$\begin{matrix} \frac{\frac{S_{ext}}{\gamma_{T}/\alpha_{T}}\partial_{S_{ext}}\left(\gamma_{T}/\alpha_{T}\right)}{\frac{S_{ext}}{\alpha_{S}}\partial_{S_{ext}}\alpha_{S}}=\frac{\frac{T}{\gamma_{T}/\alpha_{T}}\partial_{T}\left(\gamma_{T}/\alpha_{T}\right)}{\frac{T}{\alpha_{S}}\partial_{T}\alpha_{S}}(1-\frac{\frac{S_{ext}}{\gamma_{S}}\partial_{S_{ext}}\alpha_{S}}{\frac{S_{ext}}{\alpha_{S}}\partial_{S_{ext}}\alpha_{S}}) \\ \Rightarrow\frac{\partial_{S_{ext}}\left(\gamma_{T}/\alpha_{T}\right)}{\partial_{S_{ext}}\alpha_{S}}=\frac{\partial_{T}\left(\gamma_{T}/\alpha_{T}\right)}{\partial_{T}\alpha_{S}}(1-\frac{\alpha_{S}}{\gamma_{S}}\frac{\partial_{S_{ext}}\gamma_{S}}{\partial_{S_{ext}}\alpha_{S}}) \end{matrix}$ .

1. **Nutrient uptake is proportional to the number of transporters**

We consider the general case in which the nutrient uptake is proportional to the number of transporters, $\alpha_{S}=T\cdot u(S_{ext},S_{int})$, the internal nutrients usage is some function that depends on the internal nutrient concentration,$\gamma_{S}(S_{ext},S_{int})$, the transporters synthesis is some function that depends on the internal and external nutrient concentrations, $\alpha_{T}(S_{ext},S_{int})$, and the transporters downregulation is proportional to the number of transporters, $\gamma_{T}=T\cdot k_{\gamma_{T}}(S_{ext},S_{int})$.

Thus the whole system is

To investigate the criterion for robustness, we plug Eq. in into Eq. yielding

$$\begin{matrix} \frac{T\cdot\partial_{S_{ext}}\left(k_{\gamma_{T}}(S_{ext},S_{int})/\alpha_{T}(S_{ext},S_{int},T)\right)}{T\cdot\partial_{S_{ext}}u(S_{ext},S_{int})}= \\ \frac{k_{\gamma_{T}}(S_{ext},S_{int})\cdot\alpha_{T}(S_{ext},S_{int},T)-\partial_{T}\alpha_{T}(S_{ext},S_{int},T)\cdot T\cdot k_{\gamma_{T}}(S_{ext},S_{int})}{\alpha_{T}(S_{ext},S_{int},T)^{2}\cdot u(S_{ext},S_{int})}\cdot\\ (1-\frac{T\cdot u(S_{ext},S_{int})}{\gamma_{s}(S_{ext},S_{int})}\frac{\partial_{S_{ext}}\gamma_{s}(S_{ext},S_{int})}{T\cdot\partial_{S_{ext}}u(S_{ext},S_{int})})\Rightarrow\\ \Rightarrow\frac{\partial_{S_{ext}}\left(k_{\gamma_{T}}(S_{ext},S_{int})/\alpha_{T}(S_{ext},S_{int},T)\right)}{k_{\gamma_{T}}(S_{ext},S_{int})/\alpha_{T}(S_{ext},S_{int},T)\cdot(1-\frac{T\cdot\partial_{T}\alpha_{T}(S_{ext},S_{int},T)}{\alpha_{T}(S_{ext},S_{int},T)})}=\frac{\partial_{S_{ext}}u(S_{ext},S_{int})}{u(S_{ext},S_{int})}-\frac{\partial_{S_{ext}}\gamma_{s}(S_{ext},S_{int})}{\gamma_{s}(S_{ext},S_{int})} \end{matrix}$$

$\alpha_{T}\perp T$ leads to a biologically interpretable situation.

If $\alpha_{T}\perp T$ then is further simplified to

$$\frac{\partial_{S_{ext}}\left(k_{\gamma_{T}}(S_{ext},S_{int})/\alpha_{T}(S_{ext},S_{int})\right)}{k_{\gamma_{T}}(S_{ext},S_{int})/\alpha_{T}(S_{ext},S_{int})}=\frac{\partial_{S_{ext}}u(S_{ext},S_{int})}{u(S_{ext},S_{int})}-\frac{\partial_{S_{ext}}\gamma_{s}(S_{ext},S_{int})}{\gamma_{s}(S_{ext},S_{int})}$$

Using $\int\frac{dx}{x}=\log(x)+\mathrm{Const}(\mathrm{v})$ we get that Eq. becomes,

or

where is any function that depends solely on *S_int_*.

1. **Michaelian uptake**

A private case of a scenario where the uptake term has the form $\alpha_{S}=T\cdot u(S_{ext})$is if the uptake term has the standard Michaelian form with Michaelis constant *K_ext_*,

$\alpha_{S}(T,S_{int},S_{ext})=k_{cat}T\frac{S_{ext}}{S_{ext}+K_{ext}}$.

Then in order to provide global homeostasis, the following should hold

.

One of the simplest solutions that comply with Eq. is transporter degradation that also has the Michaelian form

$\gamma_{T}=k_{\gamma_{T}}T\frac{S_{ext}}{S_{ext}+K_{ext}}$ and $\alpha_{T}(T,S_{int},S_{ext})=\alpha_{T}(S_{int})$.

In this case, steady state internal nutrient concentration follows the equation

$S_{int}=\left(\frac{\alpha_{T}(S_{int})}{k_{\gamma_{S}}}\right)\left(\frac{u(S_{ext})}{k_{\gamma_{T}}(S_{ext},S_{int})}\right)$ .

And if $k_{\gamma_{T}}(S_{ext},S_{int})=\frac{k_{\gamma_{T}}}{k_{cat}}u(S_{ext})$ and $\alpha_{T}\propto const$, Eq. is reduced such that the internal nutrient concentration is a constant:

$$S_{int}=\frac{\alpha_{T}k_{cat}}{k_{\gamma_{S}}k_{\gamma_{T}}}$$

1. **Mechanism of activity-dependent downregulation**

The following system of equations describes the system schematized in Fig. 2:

where *T* is a free transporter, *C* is the complex of transporter and nutrient, *C’* is an altered conformation of the transporter nutrient complex required for transport, and *C’_mod_* is a post-translationally modified version of the transporter. *k_syn_* is the transporter synthesis rate constant; *k_on_* and *k_off_* are transporter-nutrient association and dissociation rate constants respectively; *k_con,f_* and *k_con,r_* are the forward and backward rate constants of the conformational change of the transportet, *k_cat’_* is the rate constant of nutrient uptake, *k_mod_* is the rate of modification (ubiquitinylation) of the transporter-nutrient complex; *k_int_* is the rate of modified transporter internalization.

In this system, the rate of downregulation of the transporter is $r_{td}=k_{int}\cdot C'_{mod}$ and the rate of nutrient uptake is $r_{nt}=k_{cat'}\cdot C'$. Solving the last equation at steady state shows that

$$r_{td}\propto C'\propto r_{nt}$$

This means that at steady state the transporter downregulation rate is proportional to the nutrient uptake rate.

1. **Analyzing deviations from global perfect homeostasis**

We analyzed the case in which nutrient uptake is Michaelian, internal nutrient usage is proportional to its concentration, the transporters synthesis is constant, and transporter downregulation has two terms: a basal degradation term and activity-dependent term. In ordrer to analyze it properly we define the new term $u^{*}(S_{ext})$ where $k_{cat}\cdot u^{*}(S_{ext})=u(S_{ext})$. So the meaning of $u^{*}(S_{ext})$ is a fractional occupancy of the transporter by the nutrient and it is proportioanl to the $u(S_{ext})$. Thus, $\gamma_{T}=\left(k_{\gamma_{T}}^{c}+k_{\gamma_{T}}^{a}\cdot u^{*}(S_{ext})\right)\cdot T$ where $k_{\gamma_{T}}^{c}$is basal degradation rate constant and $k_{\gamma_{T}}^{a}$is activity-dependent downregulation rate constant. The system is

where $k_{\gamma_{S}}$is the nutrient usage rate constant. In addition we assume $u^{*}(S_{ext})=\frac{S_{ext}}{S_{ext}+K_{ext}}$.

At steady state,

$$\begin{matrix} S_{int}=\frac{k_{cat}\alpha_{T}}{k_{\gamma_{S}}k_{\gamma_{T}}^{c}}\frac{u^{*}}{1+u^{*}\frac{k_{\gamma_{T}}^{a}}{k_{\gamma_{T}}^{c}}}\Rightarrow\frac{S_{int}}{S_{int,max}}=\frac{u^{*}\left(1+\frac{k_{\gamma_{T}}^{a}}{k_{\gamma_{T}}^{c}}\right)}{1+u^{*}\frac{k_{\gamma_{T}}^{a}}{k_{\gamma_{T}}^{c}}} \\ T=\frac{\alpha_{T}}{k_{\gamma_{T}}^{c}}\frac{1}{1+u^{*}\frac{k_{\gamma_{T}}^{a}}{k_{\gamma_{T}}^{c}}}\Rightarrow\frac{T}{T_{min}}=\frac{1+\frac{k_{\gamma_{T}}^{a}}{k_{\gamma_{T}}^{c}}}{1+u^{*}\frac{k_{\gamma_{T}}^{a}}{k_{\gamma_{T}}^{c}}} \end{matrix}$$

where $S_{int,max}$is the maximum of $S_{int}$values over the range of $S_{ext}$ and $T_{min}$is the minimum value of *T*.

There are two extreme cases that exemplify the behavior of the system:

First is when all transporter downregulation corresponds to activity-dependent downregulation,$k_{\gamma_{T}}^{c}\to0$, then the system reduces to

(the system described in Section III). $\frac{S_{int}}{S_{int,\max}}$is, therefore, a constant and independent of the external concentration

$$\frac{S_{int}}{S_{int,\max}}=\frac{u^{*}\cdot k_{\gamma_{T}}^{c}+u^{*}\cdot k_{\gamma_{T}}^{a}}{k_{\gamma_{T}}^{c}+u^{*}\cdot k_{\gamma_{T}}^{a}}\to\frac{u^{*}\cdot k_{\gamma_{T}}^{a}}{u^{*}\cdot k_{\gamma_{T}}^{a}}=1$$

Second is the situation when there is no transporter regulation $k_{\gamma_{T}}^{a}\to0$. Then the system reduces to:

and $\frac{S_{int}}{S_{int,\max}}$is just proportional to the Michaelis term $u^{*}(S_{ext})$

$\frac{S_{int}}{S_{int,\max}}=\frac{u^{*}\cdot k_{\gamma_{T}}^{c}+u^{*}\cdot k_{\gamma_{T}}^{a}}{k_{\gamma_{T}}^{c}+u^{*}\cdot k_{\gamma_{T}}^{a}}\to\frac{u^{*}\cdot k_{\gamma_{T}}^{c}}{k_{\gamma_{T}}^{c}}=u^{*}=\frac{S_{ext}}{S_{ext}+K_{ext}}$ .

1. **Regulation by an independent sensor that senses external nutrient concentration**

If the external nutrient concentration is measured by independent sensor, that regulates transporter downregulation, the system is

.

Thus,

$$S_{int}=\frac{k_{cat}\alpha_{T}}{k_{\gamma_{S}}k_{\gamma_{T}}}\frac{S_{ext}}{S_{ext}+K_{ext}}/\frac{S_{ext}}{S_{ext}+K_{sensor}}$$

$$\frac{S_{int}}{S_{int,\infty}}=\frac{S_{ext}/K_{ext}+K_{sensor}/K_{ext}}{S_{ext}/K_{ext}+1}$$

where $S_{int,\infty}$ is the value of internal nutrient concentration $S_{int}$ when$S_{ext}\to\infty$.

As $K_{sensor}/K_{ext}\to1$the internal nutrient concentration reaches perfect homeostasis $\frac{S_{int}}{S_{int,\infty}}\to1$.

1. **Regulation by the internal nutrient concentrations**
   1. **Transporter expression that depends on the internal nutrient concentration**

We analyzed the following system where the transporters synthesis depends on the internal nutrient concentration,

where *K_syn_* is the transporter synthesis inhibition constant.

To get the steady state solution one needs to solve

$$S_{int}=S_{0}\frac{K_{syn}}{K_{syn}+S_{int}}u(S_{ext})$$

where $S_{0}=\frac{\alpha_{T}}{k_{\gamma_{S}}k_{\gamma_{T}}^{c}}$. Then,

$$S_{int}=\frac{\sqrt{K_{syn}^{2}+4S_{0}K_{syn}\cdot u(S_{ext})}-K_{syn}}{2}$$

$$\frac{S_{int}}{S_{int,\max}}=\frac{\sqrt{K_{syn}/S_{0}}-\sqrt{K_{syn}/S_{0}+4u}}{\sqrt{K_{syn}/S_{0}}-\sqrt{K_{syn}/S_{0}+4}}$$

When the transporter regulation in not very sensitive to *S_int_*, $\frac{K_{syn}}{S_{0}}>>1$ the response is reduced to a form with constant downregulation, $\frac{S_{int}}{S_{int,max}}\approx u$and when the regulation is sensitive to the internal nutrient concentration, $\frac{K_{syn}}{S_{0}}<<1$, the response goes as the square root of the uptake, $\frac{S_{int}}{S_{int,max}}\approx\sqrt{u}$.

If we add additional *S_int_*-dependent regulation steps that correspond to the following system

the response goes towhen$\frac{K_{syn}}{S_{0}}<<1$.

The steady state solutions for *T* and *S_int_* can be obtained from the system

$\begin{matrix} S_{int}\left(\frac{K_{syn}^{n}+S_{int}^{n}}{K_{syn}^{n}}\right)=\frac{\alpha_{T}}{k_{\gamma_{T}}k_{\gamma_{S}}}\cdot u \\ T=\frac{k_{\gamma_{S}}S_{int}}{k_{cat}\cdot u} \end{matrix}$ .

In order achieve homeostasis the following statement must hold.

$$\Delta\log(T)=\log(C)-\Delta\log(u)$$

where C is some constant.

Combining Eq. and Eq. , if the $S_{int}^{n}$>>$K_{syn}^{n}$:

Where . Thus,

$$\Delta\log(T)=\log(C_{1})-\left(\frac{n+2}{n+1}\right)\Delta\log(u)$$

which approaches statement as *n* goes to infinity.

At the same time when $S_{int}^{n}$ <<$K_{syn}^{n}$:

$$\begin{matrix} \Delta S_{int}=\frac{\alpha_{T}}{k_{\gamma_{T}}k_{\gamma_{S}}}\cdot\Delta u \\ \Delta T=\frac{k_{\gamma_{S}}\frac{\alpha_{T}}{k_{\gamma_{T}}k_{\gamma_{S}}}\cdot\Delta u}{\Delta u}=C_{2} \end{matrix}$$

where $C_{2}=\frac{\alpha_{T}}{k_{\gamma_{T}}}$. Thus the system is non-homeostatic.

- 1. **Transporters downregulation that depends on the internal nutrient concentration**

We analyzed the following system where the transporters downregulation depends on the internal nutrient concentration

.

The steady state solution can be obtained by solving

$$S_{int}=S_{0}\frac{K_{deg}+S_{int}}{S_{int}}u(S_{ext})$$

where $S_{0}=\frac{\alpha_{T}}{k_{\gamma_{S}}k_{\gamma_{T}}}$. Thus,

$S_{int}=\frac{S_{0}u+\sqrt{S_{0}^{2}u^{2}+4S_{0}K_{deg}u}}{2}$ ,

$\frac{S_{int}}{S_{int,max}}=\frac{u+\sqrt{u}\sqrt{4K_{deg}/S_{0}+u}}{1+\sqrt{4K_{deg}/S_{0}+1}}$ .

When the transporter regulation in not very sensitive to *S_int_*,, $\frac{K_{deg}}{S_{0}}<<1$, the response is reduced to a form with constant transporter downregulation, $\frac{S_{int}}{S_{int,max}}\approx u$. In contrast, when the regulation is sensitive to the internal nutrient concentration, $\frac{K_{deg}}{S_{0}}>>1$, the response goes as the square root of the uptake, $\frac{S_{int}}{S_{int,max}}\approx\sqrt{u}$.

If we add gain on the *S_int_*-dependent regulation,

the response goes as the square root of the uptake, when $\frac{K_{deg}}{S_{0}}>>1$

1. **Trade-off between robustness and efficiency**

We first analyzed the case in which nutrient uptake is Michaelian, internal nutrient usage is proportional to its concentration, the transporters synthesis is constant, and the transporters downregulation has two terms, $\gamma_{T}=\left(k_{\gamma_{T}}^{c}+k_{\gamma_{T}}^{a}\cdot u^{*}(S_{ext})\right)\cdot T$ (as in section V part a),

We define robustness as

$$r=\frac{S_{int}}{S_{int,max}}=\frac{u^{*}\left(1+\frac{k_{\gamma_{T}}^{a}}{k_{\gamma_{T}}^{c}}\right)}{1+u^{*}\frac{k_{\gamma_{T}}^{a}}{k_{\gamma_{T}}^{c}}}$$

where $u^{*}=\frac{S_{ext}}{S_{ext}+K_{ext}}$. Since $0\leq\frac{k_{\gamma_{T}}^{a}}{k_{\gamma_{T}}^{c}}\leq\infty$ - $u^{*}\leq r\leq1$

We defined efficiency as the amount of nutrient transported by a single transporter over its lifetime

$$e=\frac{\alpha_{S}(S_{ext},T)/T}{\gamma_{T}(S_{ext},T)/T}=\frac{k_{cat}\cdot u^{*}(S_{ext})}{\left(k_{\gamma_{T}}^{c}+k_{\gamma_{T}}^{a}\cdot u^{*}(S_{ext})\right)}=e_{0}u\frac{1-r}{1-u^{*}}$$

where $e_{0}=k_{cat}/k_{\gamma_{T}}^{c}$. Since $u^{*}\leq r\leq1$ efficiency becomes $0\leq e/e_{0}\leq u^{*}$. When there is no activity-dependent downregulation, the robustness takes its minimal value, $r=u^{*}$ and the efficiency takes its maximal values, $e=e_{0}u^{*}$. As $k_{\gamma_{T}}^{a}/k_{\gamma_{T}}^{c}$ increases, there is a linear negative relationship between robustness and efficiency with the slope of this trade-off depending on the external nutrient concentration.

We now add to Eq. a term that accounts for transcriptional regulation of transporter synthesis that depends on *S_int_* with the inhibition constant *K_syn_*. Under the condition of $K_{syn}\ll S_{int}$ this dependence $\frac{K_{syn}}{K_{syn}+S_{int}}$ can be approximated by $\frac{K_{syn}}{S_{int}}$ which will result in the system

Then

$$S_{int}=S_{0}\sqrt{\frac{u^{*}(S_{ext})}{1+\frac{k_{\gamma_{T}}^{a}}{k_{\gamma_{T}}^{c}}u^{*}(S_{ext})}}$$

where $S_{0}=\sqrt{\frac{k_{cat}\alpha_{T}K_{syn}}{k_{\gamma_{S}}k_{\gamma_{T}}^{c}}}$.

The robustness is given by,

$r=\frac{S_{int}}{S_{int,max}}=\frac{\sqrt{u^{*}}\sqrt{1+\frac{k_{\gamma_{T}}^{a}}{k_{\gamma_{T}}^{c}}}}{\sqrt{1+u^{*}\frac{k_{\gamma_{T}}^{a}}{k_{\gamma_{T}}^{c}}}}$ .

In this case, $\sqrt{u^{*}}\leq r\leq1$.

The efficiency is given by

$$e=\frac{\alpha_{S}(S_{ext},T)/T}{\gamma_{T}(S_{ext},T)/T}=\frac{k_{cat}u^{*}}{\left(k_{\gamma_{T}}^{c}+k_{\gamma_{T}}^{a}\cdot u^{*}(S_{ext})\right)}=e_{0}u^{*}\frac{1-r^{2}}{1-u^{*}}$$

and $0\leq e/e_{0}\leq u^{*}$

Eq. is a particular case of more general form

that includes a high gain of regulation by internal nutrient concentrations.

In this case, robustness is equal to

and the efficiency is given by

$$e=\frac{\alpha_{S}(S_{ext},T)/T}{\gamma_{T}(S_{ext},T)/T}=\frac{k_{cat}u^{*}}{\left(k_{\gamma_{T}}^{c}+k_{\gamma_{T}}^{a}\cdot u^{*}(S_{ext})\right)}=e_{0}u^{*}\frac{1-r^{n+1}}{1-u^{*}}$$

**References**

1. Steuer, R., Waldherr, S., Sourjik, V., and Kollmann, M. **“Robust Signal Processing in Living Cells.”** *PLoS computational biology* 7, no. 11 (2011): e1002218. doi:10.1371/journal.pcbi.1002218
