# Supplementary figures and images for: Achieving global perfect homeostasis through transporter regulation
Source: PLoS Comput Biol. 2017 Apr 17;13(4):e1005458. doi: 10.1371/journal.pcbi.1005458 (PMC5411106; doi:10.1371/journal.pcbi.1005458)

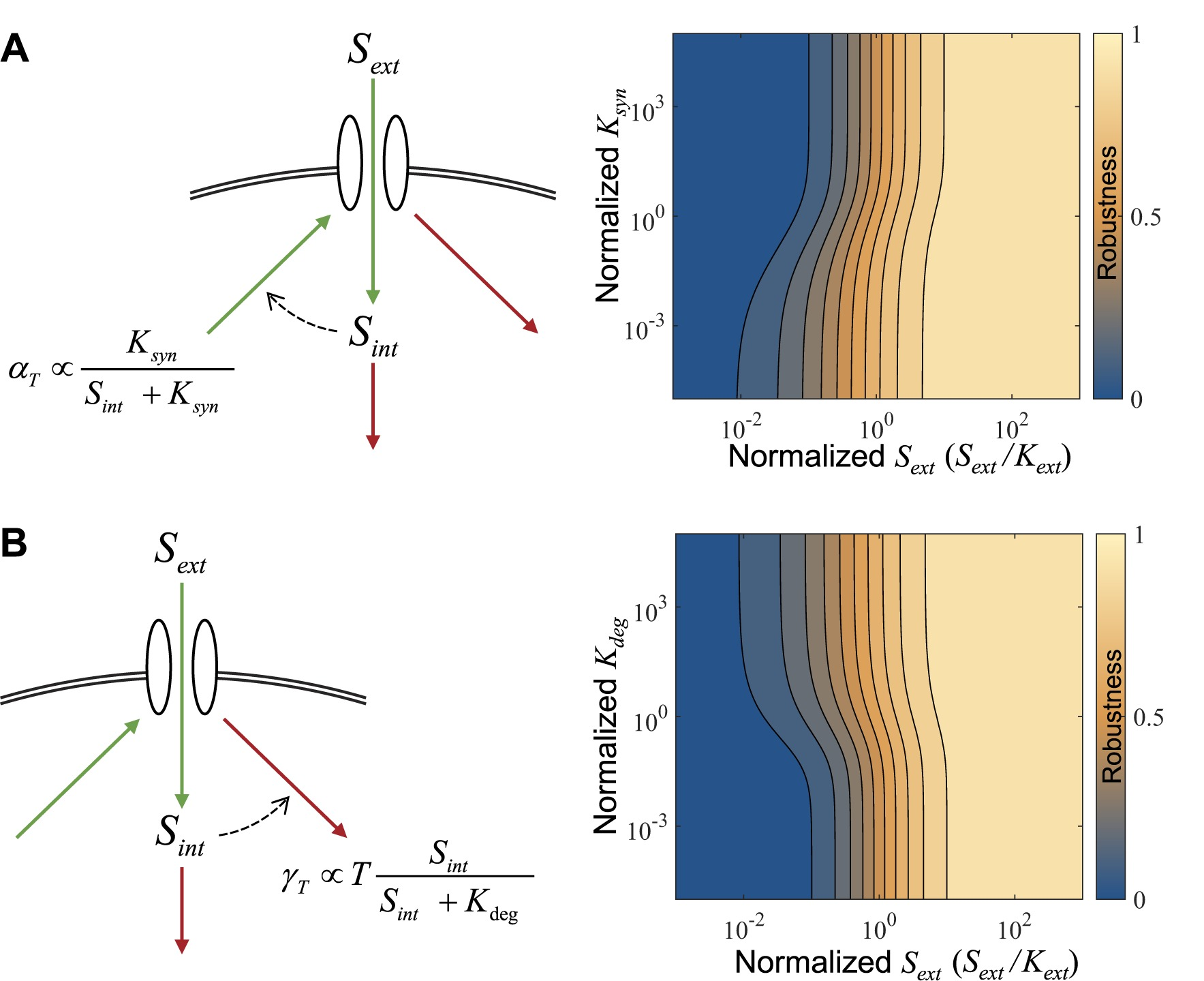

Supplement: S1 Fig — Robustness for a homeostatic system with Michaelian regulation by internal nutrient concentrations (n = 1) (A) transcriptional repression by Sint with a saturation constant of Ksyn or (B) transporter downregulation by Sint a with saturation constant of Kdeg. (TIF) [file pcbi.1005458.s002.tif]

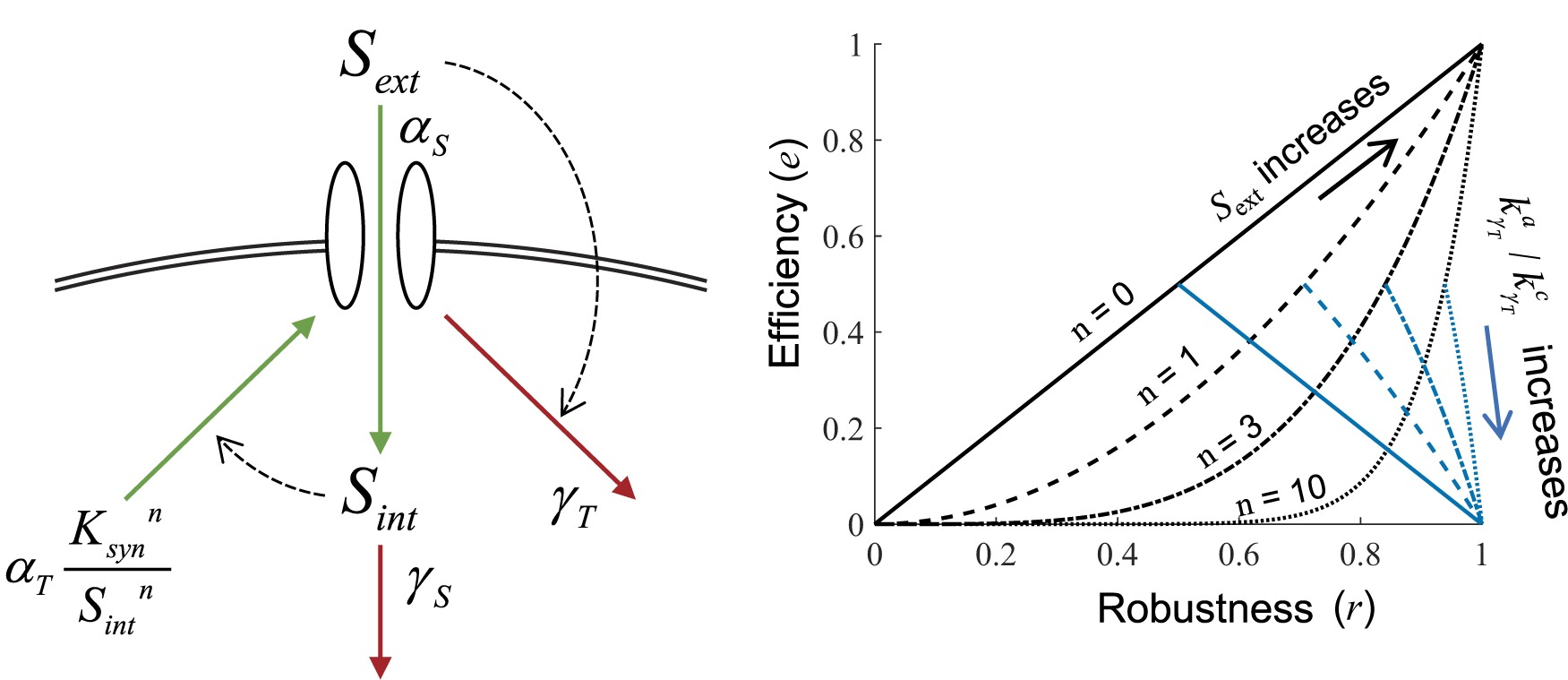

Supplement: S2 Fig — Robustness-efficiency trade-off with different orders of transporter synthesis regulation (different line style for different value of n). A system without activity-dependent transporter downregulation for a range of Sext concentrations (black). Different ratios between activity-dependent transporter downregulation and basal degradation kγTa/kγTc with constant external concentration (Sext = Kext) (blue). (TIF) [file pcbi.1005458.s003.tif]
